# Recent advances in targeted therapies and immunotherapies for triple-negative breast cancer

**DOI:** 10.3389/fonc.2026.1804620

**Published:** 2026-09-28

**Authors:** Gowhar Rashid, Deena Elsori, Zainab Naqvi, Tauqeer Zehra, Zeliha Selamoglu, Sadiya MR, Anass M. Abbas, Abubucker Peer Mohideen, Wael Hafez, Asaad Babker

**Affiliations:** 1 Department of Clinical Biochemistry, SKIMS, Srinagar, Jammu and Kashmir, India; 2 Faculty of Resillience, Deans Office Rabdan Academy, Abu Dhabi, United Arab Emirates; 3 Center for Disease Mapping and Therapeutic Research, Era University, Lucknow, India; 4 Research Metabolic Unit, Era University, Lucknow, India; 5 Department of Medical Biology, Medicine Faculty, Nigde Omer Halisdemir University, Nigde, Türkiye; 6 Department of Biology, Western Caspian University, Baku, Azerbaijan; 7 Department of Biology, Faculty of Sciences, Turkestan, Kazakhstan; 8 Suyog Institute of Allied Health Sciences, Khoja Akhmet Yassawi International Kazakh-Turkish University, Mysuru, Karnataka, India; 9 College of Applied Medical Sciences, Jouf University, Sakaka, Saudi Arabia; 10 Department of Basic Medical Sciences, College of Medicine, Prince Sattam bin Abdulaziz University, Al-Kharj, Saudi Arabia; 11 Medical Research and Clinical Studies Institute, National Research Centre, Cairo, Egypt; 12 College of Medicine and Health Science, Khalifa University, Abu Dhabi, United Arab Emirates; 13 Department of Medical Laboratory Sciences, College of Health Sciences, Gulf Medical University, Ajman, United Arab Emirates

**Keywords:** immunotherapies, molecular subtypes, patient-centric advances, targeted therapies, triple-negative breast cancer

## Abstract

This comprehensive review explores advancements in the treatment of Triple-Negative Breast Cancer (TNBC), emphasizing the transformative impact of targeted therapies and immunotherapies. Given TNBC’s aggressive nature and limited therapeutic options, this review provides an in-depth analysis of emerging strategies that aim to enhance patient outcomes. Within targeted therapies, we discuss molecular subtypes and evaluate the efficacy of PARP inhibitors, EGFR inhibitors, and PI3K inhibitors, integrating insights from clinical trials. The immunotherapy landscape is examined through immune checkpoint inhibitors, Chimeric Antigen Receptor (CAR) T-cell therapy, and novel vaccine-based approaches. Combination strategies are assessed, focusing on clinical efficacy, safety, and resistance mechanisms. Additionally, we highlight emerging therapeutic innovations, including bispecific antibodies, advanced drug delivery systems, and personalized medicine approaches. Beyond treatment modalities, the review addresses key challenges such as therapy resistance, biomarker-driven treatment selection, and the integration of Artificial Intelligence in TNBC management. Future directions emphasize ongoing clinical trials and evolving therapeutic paradigms aimed at improving survival and quality of life for TNBC patients. As the field progresses, a multidisciplinary approach integrating precision oncology, innovative therapeutics, and patient-centered care will be critical. In conclusion, this review encapsulates current breakthroughs and envisions a future where targeted and immune-based interventions redefine TNBC treatment, offering new hope for patients and clinicians alike.

## 1 Introduction

According to GLOBOCAN 2022 female breast cancer ranks second worldwide (1). Estimates project a 46% increase in the number of female breast cancer cases by 2045(2). Among Asian, African, and Latin American countries, the Republic of Korea is expected to have the highest 5-year survival rate at 89.4%, while the Islamic Republic of Iran is projected to have the lowest survival rate at 41.6%. While India has the second lowest 5-year survival rate, estimated to be 58.8% (2, 3). Breast cancer is a highly heterogeneous disease, with clinical treatment and prognosis varying significantly among patients (4, 5). Breast cancer molecular subtypes are classifications of breast cancer based on the presence or absence of specific hormone receptors and proteins. The four primary subtypes are luminal A, luminal B, human epidermal growth factor receptor 2 (HER2)-positive, and triple-negative or basal-like (TNBC). These subtypes help in determining the most effective treatment options and in predicting the disease’s behavior (4). The TNBC is a unique subtype of breast cancer defined by the lack of expression of the HER2, progesterone receptor (PR), and estrogen receptor (ER) (4). Yadav and co-workers conducted a systematic survey of stage I-III TNBC cases from the year 2010 to 2016 in both the sexes. Although low number men with TNBC were found but the 5-year survival rate was worse in men (68.8%) than women (74.8%) with TNBC (6). In a study TNBC cases represented 19.27% of breast cancer patients, with varying proportions across stages I-IV, notably higher in early stages. The study found that the overall 5-year survival rates for non-TNBC patients (86.50%) were significantly higher than those for TNBC (81.28%) patients. Survival rates decreased with advancing disease stages, showing stark differences particularly in stage III (77.48% vs. 59.27%) and stage IV (33.46% vs. 10.81%). Additionally, patients with metastatic TNBC (mTNBC) experienced worse survival outcomes than those with metastatic non-TNBC, with 5-year survival rates for lymph node-positive and -negative statuses at 13.46% and 9.75% respectively, compared to 44.54% and 33.63% in the metastatic non-TNBC group (5). The Charlson Comorbidity Index (CCI) is a widely used tool for assessing the comorbidity burden of patients, which helps to predict the risk of mortality. The index includes a list of 19 comorbid conditions, each assigned a specific weight based on its impact on mortality (7). Factors assessed by the CCI can help in understanding how comorbidities affect overall treatment outcomes, particularly in TNBC patients. In a study 2017 TNBC cases were evaluated to establish a relationship between CCI affected treatment and risk of mortality. It was found that high comorbidity increased breast-specific mortality across all disease stages. While it did not affect lumpectomy and radiation use in stage 1 breast cancer, high comorbidity was linked to reduced treatment in stages II-IV (8). It has been reported that over 85% of breast cancers that occur in individuals with BRCA1 germline pathogenic variants are TNBC. This high prevalence is due to the role BRCA1 plays in maintaining genomic stability; when it is mutated, it can lead to the development of triple-negative tumors (9). The National Comprehensive Cancer Network guidelines recommend various chemotherapy regimens for TNBC, including taxanes, cyclophosphamide, platinum agents (e.g. cisplatin and carboplatin), 5-fluorouracil, and anthracyclines (10).

## 2 Methodology and literature search strategy

A comprehensive literature search was performed to identify relevant studies on the molecular pathogenesis, targeted therapies, immunotherapies, tumor microenvironment biomarkers and emerging therapeutic strategies for TNBC. A systematic search of the published literature was performed major electronic databases: PubMed/MEDLINE, Scopus, Web of Science and EMbase. Additional searches were conducted in ClinicalTrials.gov to identify relevant ongoing and completed clinical trials from January 2025 to June 2026. The search strategy combined both Medical Subject Headings (MeSH) and free text keywords. The primary search included: “triple negative breast cancer” OR “TNBC” OR “triple negative breast neoplasm” combined with terms encompassing targeted therapies (“targeted therapy”, “PARP inhibitors”, “EGFR inhibitors”, “PI3K inhibitors”, “kinase inhibitors”, “Notch pathway inhibitors”, “antibody drug conjugates”, “ADC”), immunotherapy-related terms (“immune checkpoint inhibitors”, “PD-1”, “PD-L1”, “CTLA-4”, “CAR T-cell therapy”, “cancer vaccine”), and pathobiological terms (“tumor microenvironment”, “molecular pathogenesis”, “BRCA mutation”, “homologous recombination deficiency”). Additional terms included “drug delivery systems”, “nanocarriers”, “artificial intelligence”, “machine learning”, “biomarkers”, and “treatment resistance”. Boolean operators (AND, OR) were used to combine the search terms systematically.

Studies included original research, Phase I-IV clinical trials, systematic reviews, meta-analyses, and peer-reviewed reviews addressing TNBC biology, targeted therapies, immunotherapy, combination strategies, biomarkers, resistance mechanisms, or novel drug delivery systems with clinically or mechanistically relevant outcomes. Studies focusing on non-TNBC subtypes without TNBC-specific data, conference abstracts, editorials, letters, case reports, duplicate publications, and animal-only studies lacking translational relevance were excluded.

Studies were prioritized according to the level of evidence, with randomized controlled trials and Phase II/III clinical studies given precedence, followed by preclinical and translational research for mechanistic insights. When multiple studies addressed the same topic, preference was given to larger, more recent, and higher-impact publications, while landmark trials and FDA-approved therapies were included irrespective of publication date. Following independent screening and full-text review by two authors, a total of 248 records were identified. After duplicate removal and eligibility assessment, 174 studies were included in the final review according to the predefined eligibility criteria.

## 3 Molecular pathogenesis of tumorogenesis In TNBC-

### 3.1 Early stage (stage I)

The molecular pathogenesis of TNBC unfolds through a cascade of stage-wise oncogenic events. At the earliest stage, aberrations in the PI3K/AKT/mTOR pathway establishes a pro-oncogenic cellular environment. These aberrations regulate critical cellular processes, including cell cycle progression, metabolic reprogramming and cellular responses to growth factor and hypoxia. Overexpression of EGFR, activating mutations in PIK3CA, and loss of tumor suppressor PTEN and proline rich inositol polyphosphates collectively drive canonical PAM pathway activation (11). In a study conducted by Tu et al. demonstrated that non-canonical amplification of PI3K/AKT/mTOR pathway is mediated by the long non-coding RNA LINC01133, which activates AKT in a PI3K independent manner. Mechanistically, LINC01133 modulates mTORC2 signaling through hnRNPA2B1- dependent upregulation of PROTOR1/prr5. This regulatory mechanisms has been tightly associated with poor survival in TNBC (12). Simultaneously, dysregulation of the Wnt/β-catenin pathway compounds initiates oncogenic events. Overexpression of FzD7 and LRP6 activates β-catenin-driven transcription, with FZD7 driving ontogenesis through p63 and LRP6 facilitating early migratory behavior (13). METTL3 mediated m6A modification of FAM83D (14) and KRT17 mediated suppression of APC and AXIN1 further destabilize the β-catenin destruction complex, locking initiated cells into a transformed state (15). Tumors exhibiting nuclear β-catenin accumulation or membrane β-catenin loss were significantly more likely to exhibit as TNBC (16). Collectively, these early molecular aberrations cooperatively establish a tumor-permissive state that promotes cellular transformation, proliferative signaling, and the initiation of TNBC pathogenesis.

### 3.2 Early invasive stage (stage II)

During the early stage, activation of Notch signaling and JAK2/STAT3 signaling, and the Hedgehog (Hh) pathway synergistically enhances the malignant phenotypes. These pathways support the maintenance and expansion of breast cancer stem cell (BCSC) population. Notch1 drives tumor progression through a mitochondrial fission-positive feedback loop (17) and activates the ATR-CHK1 signaling cascade to restore S/G2 and G2/M cell cycle checkpoints thereby suppressing mitotic catastrophe arising from BRCA1 deficiency (13). Notch3, overexpressed in TNBC and significantly co-expressed with EGFR, contributes to resistance to targeted therapy (13, 18). In parallel Notch4 sustains BCSC self-renewal particularly in metastatic disease (13, 19). Within the JAK2/STAT3 pathway, HOXC10 activates the IL-6/JAK2/STAT3 cascade, and TrkA enhances STAT3 activity to promote BCSC maintenance (13, 20). VEGF-VEGFR2 further upregulates Myelocytomatosis (Myc) and Sox2 through JAK2/STAT3 signaling, coupling angiogenesis with stemness (13). Concurrently, non-canonical Hh pathway activation driven by elevated GLI1/2 downstream of PI3K-AKT, K-Ras, c-Myc, Wnt/β-catenin, TGFβ and NF- κB expand stem cell population (21) and stimulates the activation of cancer associated fibroblasts within the tumor microenvironment (TME) (13).

### 3.3 Locally advanced cancer stage (stage III)

As TNBC advances into active progression, metastatic dissemination is facilitated via convergent epithelial-mesenchymal transition (EMT) and cytoskeletal remodeling processes. Notch1 mediated EMT confers mesenchymal plasticity and enhances migratory potential (22), while SCH4 upregulation stimulates Src kinase autophosphorylation to promote metastatic dissemination through β-catenin nuclear accumulation (23). Nuclear β-catenin directly upregulates cell migration and colony formation (24), while LRP6 overexpression further sustains the invasive phenotype (13). Concurrent with metastatic progression, tumor angiogenesis is established via VEGF-VEGFR2/JAK2/STAT3 signaling. Additionally, a distinct immunomodulatory pathway involving CXCL9, which is highly expressed in M1 macrophages remodels the TME via JAK1/STAT2 signaling and MHC-II modulation (25). Under hypoxic conditions, sustained mTORC1 activity stabilizes HIF-1α to amplify the angiogenic response (11). Hh activated CAFs further contribute by secreting pro-angiogenic and pro-tumorigenic factors (13), while non-canonical GLI1/2 signaling directly facilitates neovascularization, enhancing the vascular network required for tumor expansion and metastatic dissemination (13, 26).

### 3.4 Metastatic cancer stage (stage IV)

The final pathogenic stage-drug resistance and therapeutic escape is reinforced by the same oncogenic signaling networks. Notch1 mediated ATR-CHK1 activation restores DNA damage checkpoints, thereby protecting BRCA1 deficient cells from cytotoxic stress and therapy induced mitotic catastrophe. Simultaneously Notch3-EGFR co-activation promotes resistance to targeted therapies, while Notch4 sustained BCSCs maintain a reservoir of treatment refectory cells (13, 18). Geyer et al. demonstrated that, Wnt pathway, and nuclear β-catenin accumulation directly contributes to chemoresistance (27). Moreover KRT17 inhibition can reverse doxorubicin resistance by restoring APC/AXIN1-mediated β-catenin degradation (15), while METTL3/FAM83D m6A regulation further modulates Wnt pathway sensitivity and therapeutic response (14). JAK2/STAT3 signaling anti-apoptotic survival programs that contribute to therapeutic resistance in TNBC. Suppression of this pathway by the redox enzyme WWOX and glyceryl trinitrate (28), or by the natural alkaloid piperlongumine alone or in combination with doxorubicin (17) can effectively reverse the resistant phenotype TNBC cells to treatment (13). Furthermore, Zhou et al. identified MYC as a common downstream effector of both PI3K and Wnt signaling, where it facilitates cell cycle progression and metabolic reprogramming. This functional integration may partly explain the limited efficacy of mono-pathway inhibition in stage IV cancer (29).

Together, these interdependent signaling networks define TNBC as a molecularly heterogeneous malignancy in which stage specific oncogenic activation, microenvironmental crosstalk and acquired resistance mechanisms to collectively drive its characteristically aggressive clinical behavior and poor therapeutic outcome.

## 4 Tumor microenvironment: pro- and anti-tumor effects

### 4.1 Pro TME

The TME drives profoundly immunosuppressive niche through integrated cellular, molecular and structural mechanisms (30). At the cellular level, M2 polarized tumor-associated macrophages (TAMs) upregulate the PD-L1 and CTLA-4, attenuating T-cell effector activity and driving immune suppression (31). TAMs secret matrix metalloproteinases (MMPs), notably MMP-7 and MMP-9 to degrade the ECM, thereby facilitating the endothelial invasion and endovascular formation. While cytokine-driven activation of the CCL2/AKT/β-catenin signaling pathway which further enhances cancer stem cell (CSC) maintenance and promote tumor aggressiveness (25). This convergence of macrophages signaling and CSC niche regulation constitutes a fundamental pro-tumorigenic network in TNBC (32).

Cancer associate fibroblasts (CAFs) constitute a pivotal stromal component driving pro tumor ECM remodeling through paracrine cytokine secretion (33). Co-culture experiments have demonstrated that interaction between basal like breast cancer cells and fibroblast induces high levels of interleukin-chemokine expression, including IL-6, IL-8, CXCL1, CXCL3 and TGB β (33). Among these, pro inflammatory cytokines TNFα and IL-1 β, together with their downstream pro-metastatic chemokines CXCL8 (IL-8), CCL2 (MCP-1) and CCL5 (RANTES), are expressed at significantly higher levels in basal TNBC compared with luminal-A breast cancer subtypes (34). These inflammatory mediators promote myeloid derived suppress cell (MDSC) recruitment, support CSC maintenance, and facilitate metastatic dissemination (34). TGB β further stimulates the synthesis of pro-angiogenic VEGF, elevates endothelial cell migration and promotes the requirement of ECM proteins, including fibronectin and collagen (COL) (35). At the structural level, dysregulated COL deposition and MMP mediated ECM remodeling establish a self-reinforcing pro tumor architecture (35). Excessive deposition of COL1, III, V and IX alongside upregulation of MMP-2, 9, 11 and 14 stimulates cancer invasion and metastasis through ECM degradation (36). COL-I drives EMT and upregulates MMP expression, thereby correlating with poor outcomes in breast cancer. Moreover, binding of COL IV to Discoidin domain receptors 1 (DDR1) mediates Src-dependent MMP-2/9 secretion, facilitating TNBC progression (37). These COL-integrin interactions also sustain CSC self-renewal within the remodeled niche (32). Excessive ECM deposition compressed the tumor vasculature, thereby impairing drug delivery and contributing to therapeutic resistance (38) (**Figure 1**).

**Figure 1:**
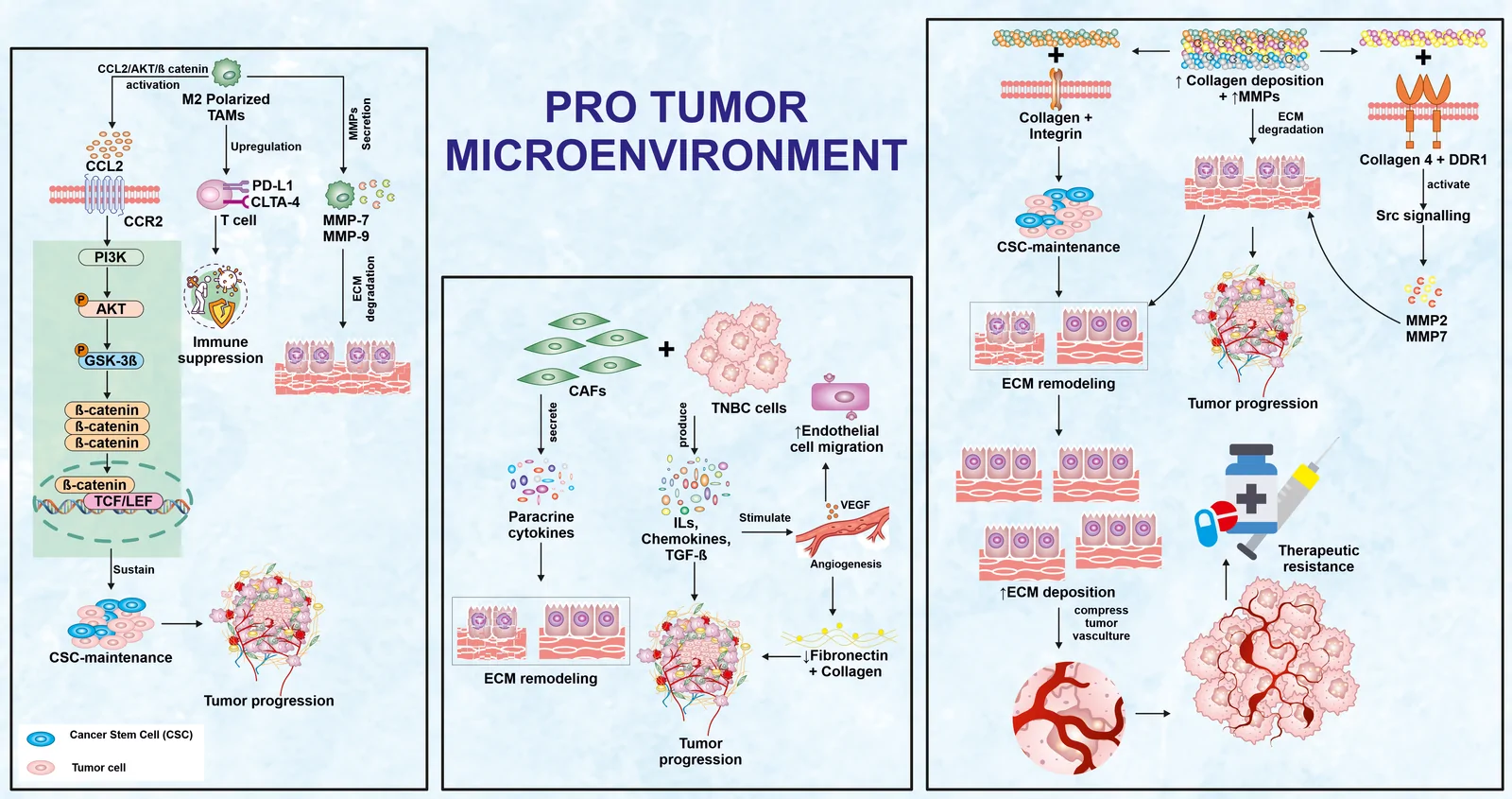
The pro-tumor TNBC microenvironment is characterized by immunosuppressive and pro-metastatic signaling. M2-polarized TAMs upregulate PD-L1 and CTLA-4 and secrete MMP-7 and MMP-9, promoting immune evasion and ECM degradation. Activation of the CCL2/AKT/β-catenin axis supports CSC maintenance and tumor progression. CAFs, together with TNBC cells, secrete IL-6, IL-8, CXCL1, CXCL3, and TGF-β, enhancing angiogenesis, ECM remodeling, MDSC recruitment. Aberrant COL deposition and increased MMP activity facilitate EMT, invasion, and metastasis, while COL IV-DDR1 signaling promotes Src-dependent MMP-2/9 secretion. Excessive ECM accumulation compresses tumor vasculature, limiting drug delivery and contributing to therapeutic resistance.

### 4.2 Anti-TME

Despite its immunosuppressive phenotypes, the TNBC TME contains significant endogenous anti-tumor regulatory mechanisms (30). CD8+ cytotoxic T- cell employ perforin, granzyme, and FasL/\TRAIL-mediated pathways to induce tumor cell apoptosis (39). Concurrently, these cells secrete IFN-γ and TNF-α, which further promote M1 macrophage polarization and elevate anti-tumor responses (39). Th1 CD4+ T cells amplify this response through IFN-γ and IL-2 secretion, upregulating MHC expression on tumor cells and facilitating CTL responses (39). Elevated tumor infiltrating lymphocytes (TIL) levels are consistently associated with improved pathological complete response (pCR) and overall survival in TNBC (30). Anti-tumor chemokines are critical mediators of immune cell trafficking (33). IFN-inducible chemokines CXCL9, CXCL10, and CXCL11 signaling through CXCR3, facilitate the recruitment of effector T cells to the TME. Moreover, TNBC patients exhibiting elevated CXCL9/10, and CXCR3 expression exhibit extended survival following pemobrolizumab treatment (40). CXCL9 and CXCL10 further induce a self-reinforcing feedback loop accelerating effector activity of both CD4+ and CD8+ T cells while suppressing the immunoregulatory protein TIM-3 (41). IL-30 also maintains breast CSC populations via CXCL10 and IL-23 autocrine loops, reflecting the dual immunomodulatory role of cytokine networks in TNBC (32). The pro-inflammatory IL-12 further stimulates anti-tumor immune responses. IL-12 treatment significantly reduces tumor progression, increases intratumoral CD8+ T cells and IFN-γ level and decreases the abundance of M2 macrophages and MDSCs in TNBC models (42).

Structurally, COL III exhibit tumor restricted properties in breast cancer microenvironment. Elevated COL III expression maintains a restrictive ECM architecture that negatively correlates with tumor aggressiveness (43). Col-derived matrikines additionally suppress CSC proliferation and survival, though their precise roles in TNBC remain under active investigation (32) (**Figure 2**). Collectively, these anti-tumor mechanisms including T-cell effectors, the IFN-γ/CXCR3 chemokines signaling, IL-12, IL-2 immunostimulatory interleukins and structurally protective COL III define actionable targets for transforming a “cold” TNBC TME into an immunologically “hot” state. This can be achieved through immune checkpoint inhibition, macrophage reprogramming strategies, CAF targeted therapies, and combination immunoengineering approaches (44, 45).

**Figure 2:**
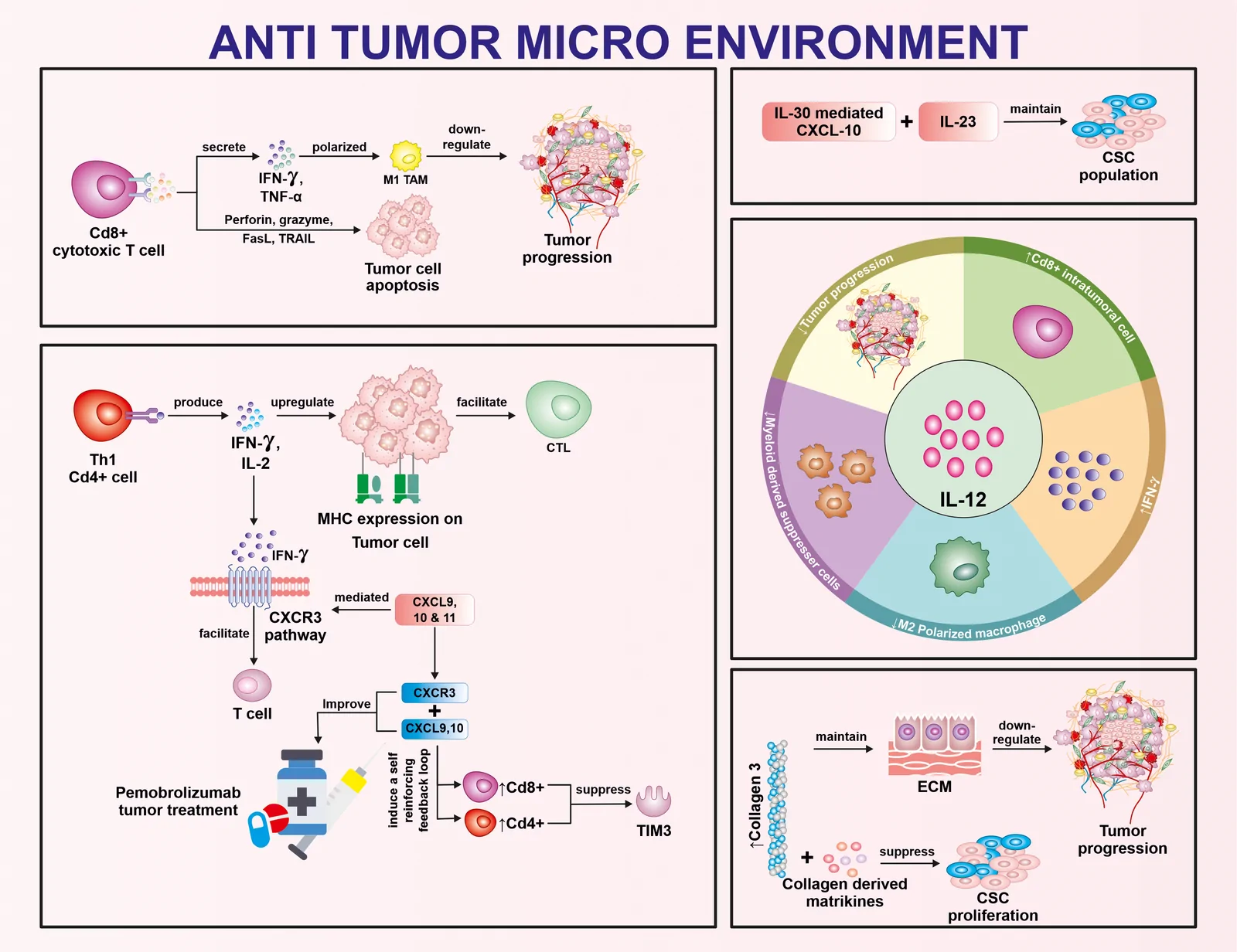
The anti-tumor microenvironment is driven by cytotoxic and immune-activating responses. CD8+ T cells induce tumor cell apoptosis through perforin, granzyme, FasL, and TRAIL pathways, while Th1 CD4+ T cells enhance anti-tumor immunity via IFN-γ and IL-2 secretion. CXCL9, CXCL10, and CXCL11 signaling through CXCR3 recruits effector T cells and correlates with improved response to pembrolizumab. IL-12 promotes anti-tumor activity by increasing intratumoral CD8+ T cells and IFN-γ levels while reducing M2 macrophages and MDSCs. Elevated COL III expression maintains a restrictive ECM architecture associated with reduced tumor aggressiveness, and COL-derived matrikines suppress CSC survival and proliferation. Collectively, these mechanisms represent potential therapeutic targets for converting immunologically “cold” TNBC tumors into “hot” immune-responsive tumors.

## 5 Importance of targeted therapies and immune response

The standardized treatment regimens for TNBCs remain under discussion among scientists and clinicians. Evidence supports sensitivity to fluoropyrimidines, platinum compounds, and anthracyclines (10). Hormone therapy and anti-HER-2 agents have shown promising results in other subtypes of breast cancer but absence of targetable molecules in TNBC limits the efficacy of other drugs (10). Recent advances in characterizing the TNBC microenvironment have enabled development of novel targeted agents (46). Among these, immunotherapy particularly immune checkpoint blockade has emerged as a promising strategy, demonstrating potential to enhance antitumor immune responses and improve survival outcomes in TNBC patients (47). Consistent with these advances, immune checkpoint inhibitors (ICIs) have emerged as an important therapeutic strategy in TNBC. Pembrolizumab remains approved in selected TNBC populations, whereas the previous accelerated approval of atezolizumab for PD-L1-positive metastatic TNBC was later withdrawn after subsequent trial findings failed to confirm clinical benefit (48–50). Additionally, poly ADP-ribose polymerase (PARP) inhibitors have demonstrated a greater clinical benefit in patients with BRCA mutated genes (51). Similarly, antibody-drug conjugates (ADC) offer a re-evaluation of cytotoxic agents previously limited by toxicity (52). Despite these advances, the successful translation of promising preclinical findings into consistent and durable clinical benefits remains a significant challenge. Therefore it is essential to distinguish evidence-based approved therapies from investigational and preclinical strategies.

### 5.1 PARP inhibitors

#### 5.1.1 Rationale and mechanistic basis

Among targeted therapies, PARP inhibitors represent the most biologically and clinically validated strategy in germline BRCA1/2 mutated TNBC, exploiting the principle of synthetic lethality. BRCA1/2, and other homologous recombination repair proteins are essential for the repair of double strand breaks; their loss creates dependency on base excision repair mediated by PARP1/2 (53–55). PARP1 and PARP2 use NAD+ to add poly (ADP-ribose) chains to itself (autoPARylation) (**Figure 3**). This process facilitates the recruitment of DNA repair proteins such as DNA ligase III, DNA polymerase β, and XRCC1 (51, 54). After recruiting repair factors, PARP1 is released from the DNA. The poly (ADP-ribose) chains are subsequently degraded by glycohydrolases or ADP-ribose hydrolase 3 to complete repair (51, 54). Additional double-strand break repair factors-ATM (ataxia telangiectasia mutated) and Fanconi anemia proteins are also critical for genomic stability (51, 56). Approximately 60% to 80% of breast cancers in BRCA1 mutation carriers are classified as TNBC (51). Some sporadic TNBCs exhibit “BRCAness” homologous recombination deficiency via epigenetic and somatic mechanisms which broaden the potential treatment population, though reliably identifying this group prospectively remains unresolved (51).

**Figure 3:**
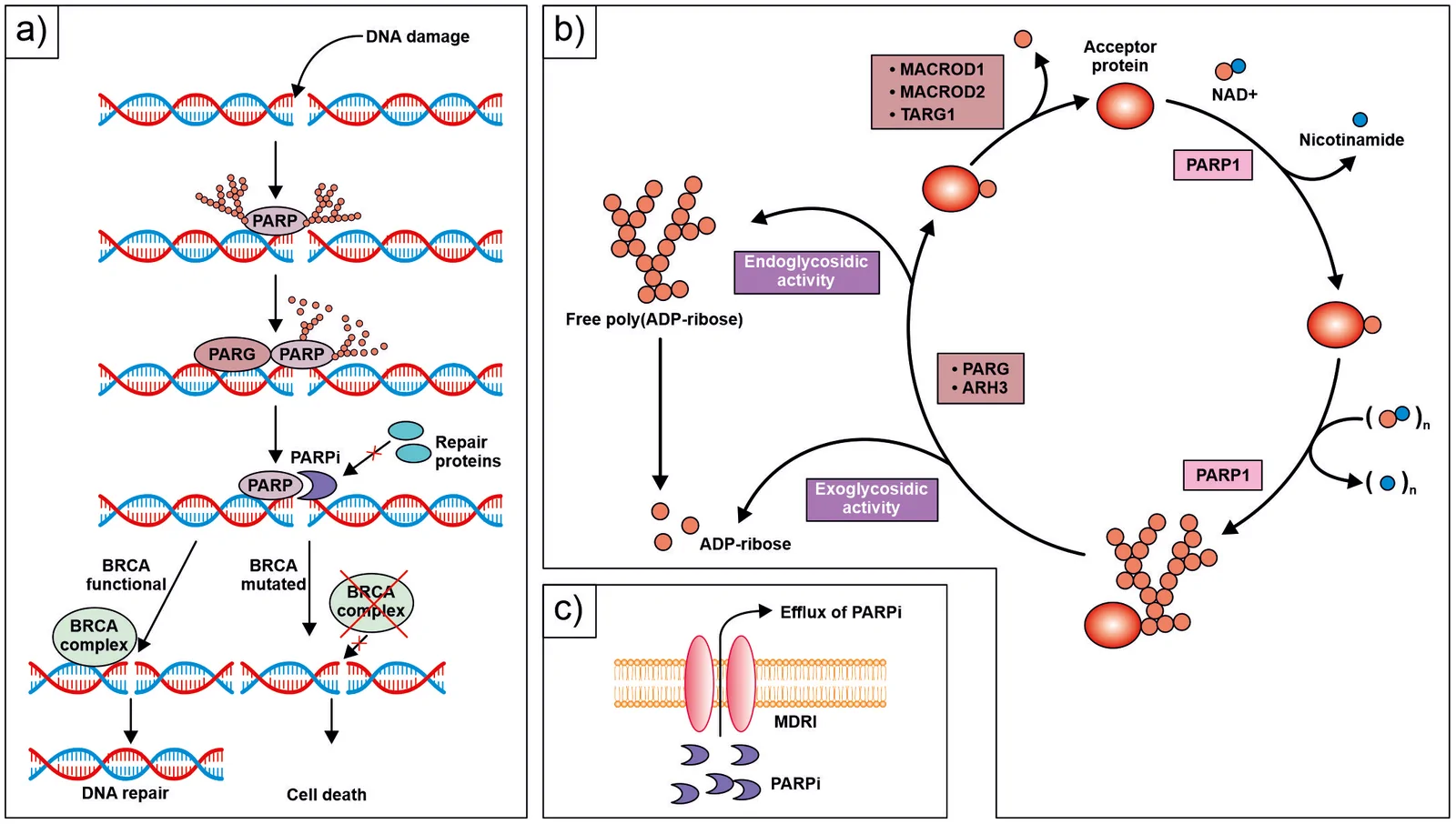
Role of PARP **(A)** PARP detects single strand DNA breaks and promotes DNA repair via PARylation. In BRCA-deficient cells, PARP inhibitor block this repair pathway, leading to impaired homologous recombination cell death. **(B)** PARP catalyzes mono (ADP-Ribosylation) (MARylation) or poly (ADP-ribosylation) (PARylation) using NAD +. MARylation is reversed by MACROD1, MACROD2, and TARG1, while PAR chains are degraded by PARG and ARH3. **(C)** MDR1 (ABCB1)-mediated efflux of PARP inhibitors contributes to therapeutic resistance in TNBC.

#### 5.1.2 Approved drugs: clinical evidence and limitations

Talazoparib was evaluated in the randomized phase III EMBRACA trial. The study demonstrated a statistically significant improvement in median PFS of 8.6 vs 5.6 months compared to standard chemotherapy group. However, the trial was not powered to detect an overall survival benefit because overall survival was not the primary endpoint (55, 56). Despite a higher rate of hematologic adverse events, particularly thrombocytopenia and anaemia patient-reported quality-of-life outcomes were reportedly superior to standard chemotherapy, a nuanced finding important to treatment decision-making. In the neoadjuvant NEOTALA trial (single-arm, phase II), talazoparib yielded pCR rates of 45.8% in evaluable and 49.2% in intent-to-treat TNBC patients with germline BRCA mutations (55). These results are encouraging but require cautious interpretation given the absence of a direct chemotherapy comparator. Litton et al. corroborated these findings, reporting pCR and residual cancer burden outcomes in germline BRCA1/2-positive early-stage TNBC. These results further validate the neoadjuvant activity of talazoparib in this molecularly selected patient population (57).

Olaparib’s adjuvant efficacy was established through the landmark OlympiA trial, a well-designed, placebo-controlled phase III study in early-stage HER2-negative breast cancer with germline BRCA1/2 mutations following neoadjuvant chemotherapy. At three years, olaparib achieved significantly improved invasive disease-free survival (85.9% vs. 77.1%) and distant disease-free survival (87.5% vs. 80.4%) compared to placebo (55, 58). These clinically meaningful outcomes justify olaparib’s current regulatory approval. Longer follow-up data from the OlympiA trial have subsequently confirmed an overall survival benefit, strengthening the evidence base for adjuvant olaparib in this population (58). Nevertheless, careful adherence to the high-risk eligibility criteria established in the OlympiA trial is essential. These criteria include prior neoadjuvant chemotherapy with residual disease or other features of high-risk early-stage disease. Restricting treatment to this population helps maximize clinical benefit while avoiding unnecessary toxicity in lower-risk patients (58).

An important area of ongoing investigation is the BrighTNess trial, which evaluated the addition of the PARP inhibitor veliparib plus carboplatin, or carboplatin alone, to standard neoadjuvant paclitaxel in TNBC. While the initial results showed that adding carboplatin significantly improved pCR rates, long-term follow-up data (median 4.5 years) revealed that veliparib did not provide additional event-free survival benefit beyond carboplatin alone (24, 59). This is a critical negative finding: despite promising pCR improvements, veliparib failed to translate these into improved long-term outcomes. The BrighTNess data serve as an important caution against equating pCR improvements with durable survival benefit. The findings demonstrate that increased pCR rates do not necessarily translate into durable survival outcomes, a distinction that is critical for the appraisal of neoadjuvant PARP inhibitor trials. Additionally, a small pilot study of lapatinib combined with veliparib in metastatic TNBC lacking BRCA1/2 mutations showed some anti-tumor signals but remained underpowered for definitive conclusions, with modest clinical translation (60). These findings indicate that, although the dual therapy approach warrants further investigation in TNBC, additional adequately powered clinical studies are required to establish its therapeutic value. The clinical trial was registered at ClinicalTrials.gov on 12 September 2014 (60, 61).

#### 5.1.3 Resistance mechanisms and combination strategies

Resistance to PARP inhibitors arises via reversion mutations restoring homologous recombination, drug efflux upregulation, and activation of alternative survival pathways necessitating combinations to sustain efficacy. Salt-Inducible Kinase 2 (SIK2) inhibitors (ARN3236 or ARN3261) combined with olaparib significantly enhanced growth inhibition in TNBC cell lines. Given that ARN3261 was effective regardless of BRCA1 status, the combination of SIK2 and PARP inhibitors could be considered for a wider range of TNBC and ovarian cancer subtypes (62). The CDK inhibitor dinaciclib combined with niraparib re-sensitised PARP inhibitor-resistant TNBC cells. It achieves this through downregulation of MYC expression, EMT, and cancer stem-like phenotypes (51). Similarly, in olaparib-resistant cells, inadequate DNA damage caused by olaparib led to activation of the Wnt signaling pathway and upregulation of MYC. The overexpression and hyperphosphorylation of β-catenin at Ser675 are found to be associated with olaparib resistance. The combination of olaparib and palbociclib (CDK4/6 inhibitor) was found effective in treating BRCA mutant/TNBCs that exhibit high Wnt signaling and MYC expression, particularly in cases resistant to PARP inhibitor monotherapy (63). SR-4835 (CDK12/13 inhibitor) synergises with cisplatin, doxorubicin, and PARP inhibitors in TNBC cells by downregulating DNA damage response proteins and augmenting γ-H2AX-mediated apoptosis (64). 53BP1 and RIF1 inhibit BRCA1 recruitment to double-strand breaks, with CDKs integral to regulating these competing repair pathway choices (51, 56). Ceralasertib (AZD6738, ATR inhibitor) combined with olaparib achieved complete tumor regression in BRCA2-mutant xenograft models, with complete regression even in BRCA wild-type models at higher doses (65). However, xenograft models are poor predictors of human benefit, and these combinations await clinical validation.

Epigenetic modulation offers additional sensitisation strategies: HDAC inhibitors (vorinostat, panobinostat) induce a BRCAness phenotype in BRCA1-mutant cells, enhancing PARP inhibitor sensitivity (51). The DNA methyltransferase (DNMT) inhibitor decitabine and next generation agent GSK3685032 are under clinical investigation for breast cancer (55). POLθ inhibition, HSP90 inhibition, c-Met inhibition (crizotinib, foretinib), and AKT inhibitors all show preclinical synergy with PARP inhibitors through distinct mechanistic rationales (51, 54). These approaches remain at the preclinical stage. Therefore, the field must prioritise the most promising combinations for clinical investigation rather than accumulating further preclinical data without translational follow-through.

#### 5.1.4 Immunomodulatory effects

An underappreciated dimension of PARP inhibitor biology is immunomodulation. In a study, BRCA1-associated TNBCs showed significantly greater immune infiltration including CD68+/CD163+PD-L1+ macrophages and T-cells expressing PD-1 (CD3^+^CD8^+^, CD3^+^CD4^+^, CD8^+^GrB^+^, CD4^+^FoxP3^+^, CD8^+^PD-1^+^, CD4^+^PD-1^+^ expressing cells), while BRCA-WT tumors had fewer immune cells (66). Olaparib exerts dual immunological effects. On one hand, it promotes anti-tumor macrophage phenotypes (CD80, CD86, CD40 upregulation) and activates the cGAS/STING pathway, generating IFNβ, CCL5, and CXCL10 that recruit CD8+ T cells in BRCA1-deficient cells (66, 67). On the other hand, olaparib also enhances immune-suppressive features, evidenced by increased PD-L1 and CSF-1R expression. This dual effect could contribute to a complex TME where both anti-tumor and immune-suppressive mechanisms are simultaneously active (66). This dual modulation provides biological rationale for PARP inhibitor/ICI combinations but also explains why simple mechanistic hypotheses may not translate to clinical benefit. Notably, Halder et al. (68) have begun to characterize how rucaparib treatment affects TME composition and PD-L1 expression in TNBC. The study provides early clinical evidence that PARP inhibition can modulate the immune microenvironment in human tumors. These findings represent an important advance beyond the predominantly preclinical mechanistic evidence available to date.

### 5.2 EGFR inhibitors

#### 5.2.1 The persistent gap between biological rationale and clinical efficacy

EGFR is overexpressed in a substantial proportion of TNBCs and is associated with aggressive disease and poor prognosis (69). Despite this apparent biological rationale, EGFR-targeted therapies have consistently underperformed in TNBC clinical trials. This discrepancy highlights that EGFR overexpression alone is insufficient to predict therapeutic benefit. The findings underscore the limitations of inferring clinical relevance solely from biomarker expression data. Two principal modalities; monoclonal antibodies (cetuximab, panitumumab) and small-molecule TKIs (gefitinib, erlotinib), have been extensively evaluated, largely with disappointing single-agent results (69, 70). Mechanisms of intrinsic EGFR inhibitor resistance include activation of alternative receptor tyrosine kinase pathways, downstream RAS/MAPK and PI3K/AKT signaling independent of EGFR and the adaptive signaling rewiring that characterizes TNBC.

Cetuximab + carboplatin or cisplatin has demonstrated an improvement in pCR rates in mTNBC patients. This suggests that chemotherapy may be required to realize additive EGFR targeting benefit (53, 55). The TBCRC 001 randomized phase II trial formally evaluated cetuximab with or without carboplatin in stage IV TNBC. Cetuximab monotherapy yielded a response rate of only 6%. The addition of carboplatin improved the response rate to 17%, indicating that is likely essential for achieving therapeutic benefit of EGFR-directed antibodies in TNBC (71). Similarly Nabholtz et al. evaluated panitumumab with anthracycline/taxane based chemotherapy in operable TNBC. The observed benefit was concentrated in biologically defined subgroups, highlighting the importance of interacting predictive biomarker identification with EGFR inhibitor programs (72).

A particularly informative clinical experience comes from the phase I study of erlotinib combined with metformin in metastatic TNBC (73). The combination was well tolerated but demonstrated limited antitumor activity. These findings indicate that even biologically rational combination strategies targeting both EGFR and metabolic pathways may fail to achieve meaningful clinical benefit in unselected TNBC populations. Similarly, bendamustine combined with erlotinib caused severe and prolonged lymphopenia in mTNBC patients without commensurate benefit (74), a cautionary example of unacceptable toxicity in the EGFR inhibitor combination space. These clinical experiences collectively reinforce that EGFR inhibition in TNBC requires both rigorous patient selection and an appropriate therapeutic context. The consistent failure of EGFR TKI monotherapy across multiple well-designed studies should discourage further unselected single-agent evaluation and instead mandate prospective biomarker stratification in any future EGFR inhibitor trials in TNBC.

#### 5.2.2 Combination strategies

Clinically, cetuximab with carboplatin or cisplatin can significantly enhance pathologic complete response rates in mTNBC patients (53, 55). However, the gefitinib/carboplatin/docetaxel combination has shown synergistic cytotoxicity preclinically (53). CDK12/13 inhibition represents a promising tool for sensitising TNBC to EGFR inhibitors, potentially improving patient outcomes (**Figure 4**). It downregulates MYC and MCL-1 and prevents cap-dependent translation through 4E-BP1 dephosphorylation, an effect dependent on CNOT1-mediated mRNA stability (52). A cetuximab-SNS-032 (CDK inhibitor) ADC limited tumour growth in EGFR-positive TNBC models without significant toxicity (52). This targeted strategy may provide a promising treatment for aggressive breast cancer subtypes, especially those resistant to standard therapies (52). Dakroub and co-workers evaluated the therapeutic potential of the PRMT5 inhibitor EPZ015938 in combination with HER2-targeted agent’s tucatinib and neratinib. Both combinations demonstrated synergistic interactions that effectively reduced cell proliferation in HCC1954 and MDA-MB-453 cells. High mRNA levels of PRMT5 were also found to be associated with poor prognosis in TNBC (75). Concurrent EGFR and IKK/NF-κB inhibition (gefitinib + IKK16) synergistically downregulated NF-κB target genes and upregulated tumor suppressors FABP3, FADS2, and FDFT1 (76). Doxazosin, an alpha-1 adrenergic blocker, exhibited EGFR binding affinity of -9.004 kcal/mol in molecular docking studies. It also suppressed CSC-like traits, reducing CD44high/CD24low subpopulations, ALDH1 activity, mammosphere formation and tumor growth preclinically (77). Computational binding data and xenograft results constitute early-stage evidence, and clinical translation cannot be assumed. The consistent absence of prospective clinical validation across most EGFR combination strategies represents a significant gap.

**Figure 4:**
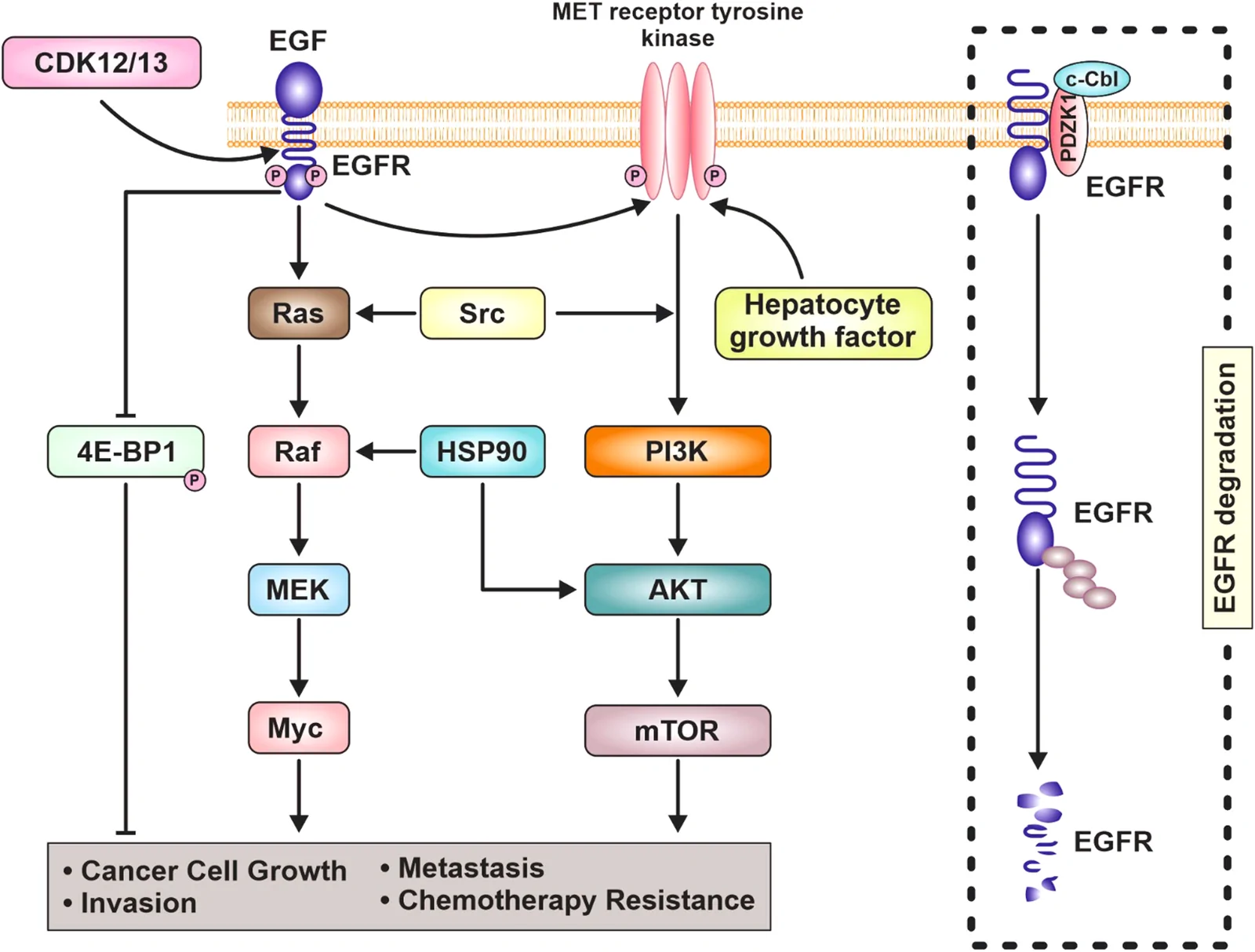
EGFR overexpression in TNBC activates the RAS/RAF/MEK/ERK and PI3K/AKT/mTOR pathways, promoting tumor growth, invasion, metastasis and chemotherapy resistance. Functional interaction between EGFR and MET signaling further enhances oncogenic acitivity. Resistance to EGFR-targeted may arise through EGFR mutations, alternative pathway or CDK12/CDK13 dysregulation. PDZK1 promotes EGFR degradation by facilitating c-Cbl mediated ubiquitination and lysosomal degradation of EGFR.

#### 5.2.3 Molecular mediators of resistance

PDZK1 promotes EGFR degradation via interaction with c-Cbl and suppress EGFR phosphorylation. Its downregulation in erlotinib-resistant TNBC has been associated with lymph node metastasis and reduced survival outcomes. These findings suggest that PDZK1 may serve as both a prognostic biomarker and a potential target for sensitizing TNBC to EGFR-directed therapies (78). BCAP31, an independent prognostic predictor for overall and disease-free survival. It regulates EGFR Y845 phosphorylation and RAB11-dependent EGFR recycling; disrupting the BCAP31–RAB11 axis may improve EGFR-targeted therapy efficacy (70). Neratinib combined with everolimus or trametinib enhanced apoptosis and tumor growth suppression relative to neratinib alone, maintaining sustained pHER2/pEGFR inhibition and further decreasing the proliferation marker Ki-67 (79). Photodynamic therapy also shows synergistic activity with EGFR inhibitors in preclinical breast cancer models (80).

### 5.3 PI3K/AKT/mTOR inhibitors

#### 5.3.1 Pathway significance and evidence landscape

The PI3K/AKT/mTOR pathway is among the most frequently dysregulated in TNBC and contributes to chemoresistance and malignant progression (**Figure 5**) (81). Clinical translation has been uneven. First-generation mTOR inhibitors everolimus and sirolimus are FDA approved for breast cancer but primarily in ER-positive disease. PI3K inhibitors specifically targeting TNBC remain in early phase development (55). A phase II trial of everolimus combined with carboplatin in mTNBC (82) showed modest activity, with 9 to 28 evaluable patients achieving clinical benefit. The limited efficacy reinforces that mTOR monotherapy or empirical chemotherapy combinations are insufficient in unselected TNBC, and patient stratification by PIK3CA/PTEN/AKT alterations is essential.

**Figure 5:**
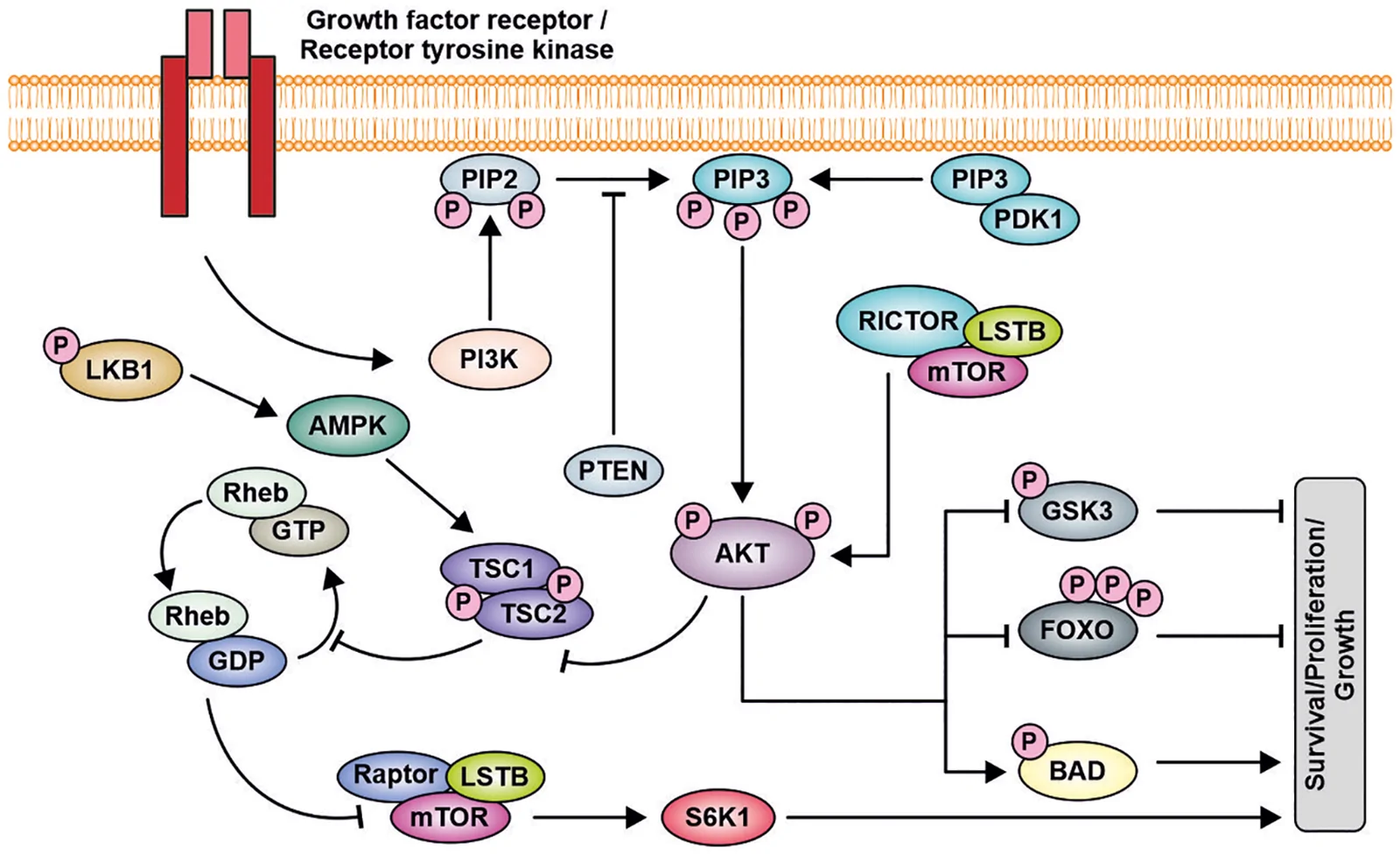
The PI3K/AKT/mTOR pathway is a major regulator of cell survival, growth, and metabolism in TNBC. Activation of receptor tyrosine kinases stimulates PI3K-mediated conversion of PIP2 to PIP3, promoting recruitment and activation of AKT by PDK1 and mTORC2. Activated AKT phosphorylates downstream targets including TSC1/2, GSK3, FOXO, BAD, and S6K1, thereby enhancing cell proliferation, survival, and tumor progression. PTEN negatively regulates this pathway by dephosphorylating PIP3, whereas Rheb-mediated activation of mTORC1 further amplifies oncogenic signaling.

#### 5.3.2 Clinical evidence for AKT and PI3K inhibitors

The most compelling clinical evidence for AKT inhibition in TNBC comes from the double-blind, randomized phase II AKT trial of capivasertib + paclitaxel vs placebo + paclitaxel as first line therapy in mTNBC. Capivasertib significantly extended median overall survival (19.1 vs. 12.6 months) and PFS (5.9 vs. 4.2 months) (83). Crucially patients with PIK3CA/AKT1/PTEN-altered tumors experienced a median PFS benefit (9.3 vs. 3.7 months), while the improvement in unselected patients was more modest reinforcing that biomarker based selection is critical (83). The subsequent phase III CAPItello-290 trial evaluating capivasertib + paclitaxel in first line mTNBC confirmed this biomarker interaction, with the PIK3CA/AKT1/PTEN-altered subgroup deriving the greatest PFS benefit. The EAY131-Y substudy of NCI-MATCH further corroborated single-agent capivasertib activity in AKT1 E17K mutated metastatic tumors (55).

Alpelisib, an α-subunit-specific PI3K inhibitor approved for PIK3CA mutated ER+/HER2- breast cancer (84), demonstrated a 30% overall response rate (ORR) and improved PFS in pretreated ER+ patients with PIK3CA mutations (85). A phase I study alpelisib combined with capivasertib in HER2- negative metastatic breast cancer demonstrated manageable toxicity at the recommended phase II dose (86). However, this study suggests feasibility of this combination but without TNBC-specific efficacy data. A phase I/II study of alpelisib combined with nab-paclitaxel in HER2-negative metastatic breast cancer (87) reported a disease control rate of 54%, with some signal in PIK3CA mutated patients within the TNBC-specific subgroup.

The PI3K/AKT inhibitors (ipatasertib and taselisib) induce autophagy in various breast cancer models, particularly in PI3K/AKT resistant TNBC cells. Inhibition of autophagy with chloroquine enhances the anti-cancer effects of PI3K/AKT inhibitors, both *in vitro* and *in vivo*, and improves response to taxane-based chemotherapy (88). The LOTUS trial of ipatasertib + paclitaxel vs. placebo + paclitaxel in first line mTNBC (89). The combination showed a trend toward improved PFS (6.2 vs. 4.9 months) that was more pronounced in PTEN-low tumors, providing an earlier randomized signal for AKT inhibition in TNBC. Importantly, the LOTUS results were considered hypothesis generating rather than practice changing, illustrating the need for well powered, biomarker stratified phase III trials. The PTK7 targeted ADC PF-06647020 yielded a 21% ORR in TNBC with moderate to high PTK7 expression (90). These findings support the broader potential of ADC based approaches within this signaling landscape. SF1126 (PI3K inhibitor) combined with gefitinib induced apoptosis in TNBC cells by blocking the EGFR-PI3K-AKT-mTOR pathway (55), but clinical data remain absent.

### 5.4 Other kinase inhibitors

#### 5.4.1 IGF1R inhibitor: clinical failures contextualize preclinical optimism

A phase III study of ganitumab added to endocrine therapy in metastatic HR+/TNBC found no PFS improvements and negatively impacted overall survival, with frequent hyperglycaemia (69). These clinical results stand in contrast to preclinical tumor regression when ganitumab was combined with PI3K inhibitor BYL719 in MCF-7 Xenografts (69) a recurring translational gap in TNBC drug development. Yee et al. further evaluated ganitumab was combined with metformin and standard neoadjuvant therapy in stage 2/3 breast cancer, including TNBC (91). While the combination was feasible, no significant improvement in pCR or biomarker defined outcomes was observed, further limiting clinical interest for IGF1R blockade in breast cancer (91). The interaction between IGF-1 and the Wnt/β catenin pathway represents a potential therapeutic target based on preclinical rationale. However, despite reduced IGF-1 levels and CSC proliferation following β catenin inhibition (69), this has not produces clinical benefits. Targeting dormant cancer cell population via MAPK inhibitors (staurosporine, trametinib) combined with chemotherapy reduced dormant cell fraction from 48.83% to 22-36% in gene expression studies (46). However, clinical translation of dormancy targeting faces substantial challenges in patient identification and trial design.

#### 5.4.2 MLK3 inhibitors; emerging preclinical evidence and prospects for clinical translation

Mixed-lineage kinase 3 (MLK3) is a serine/threonine kinase of the MAP3K family, regulates TNBC proliferation, survival invasion and metastasis and governs the ERK-1/MMP signaling pathway (92). The NGF (nerve growth factor)/TrkA pathway plays a vital role in modulating MLK3 activity. The TNBC cell lines that are unresponsive to MLK3 inhibitors exhibit low TrkA expression. And with the overexpression of TrkA, sensitivity to MLK3 inhibition is found to be restored, suggesting that the efficacy of MLK3 targeting is linked to TrkA expression levels (93). Both α-thrombin and PAR1 agonists activate MLK3, which in turn mediates JNK activation in TNBC cell lines (94). Therefore, MLK3 inhibitors could be suggested to patients with high TrkA, and JNK expression. Also, it can be administered with α-thrombin and PAR1 inhibitors for effective treatment (**Figure 6**). The TrkA/STAT3 interaction provides further mechanistic context. TrkA phosphorylates STAT3 to promote BCSC maintenance in TNBC (20), creating a convergent signaling node that may amplify TrkA dependent MLK3 pathway activity. Furthermore, dual TrkA and JAK2 pathway inhibition using entrectinib and pacritinib suppressed growth and metastasis in HER2 positive and TNBC preclinical models (95). These findings indicate that co-targeting TrkA related pathway may yield greater benefit than single agent MLK3 inhibition. Supporting this concept, CEP-1347, an MLK3 inhibitor, demonstrated antitumor activity in TNBC cell and xenograft models through suppression of the MLK3/JNK signaling pathway. These studies reported reduced tumor growth and identified TrkA as a potential predictive biomarker associated with responsiveness to MLK3-targeted therapy, further supporting MLK3 as a therapeutic target in TNBC (92, 93, 96). However, current evidence remains limited to preclinical studies, and the clinical safety and efficacy of MLK3 inhibitors in TNBC have not yet been established.

**Figure 6:**
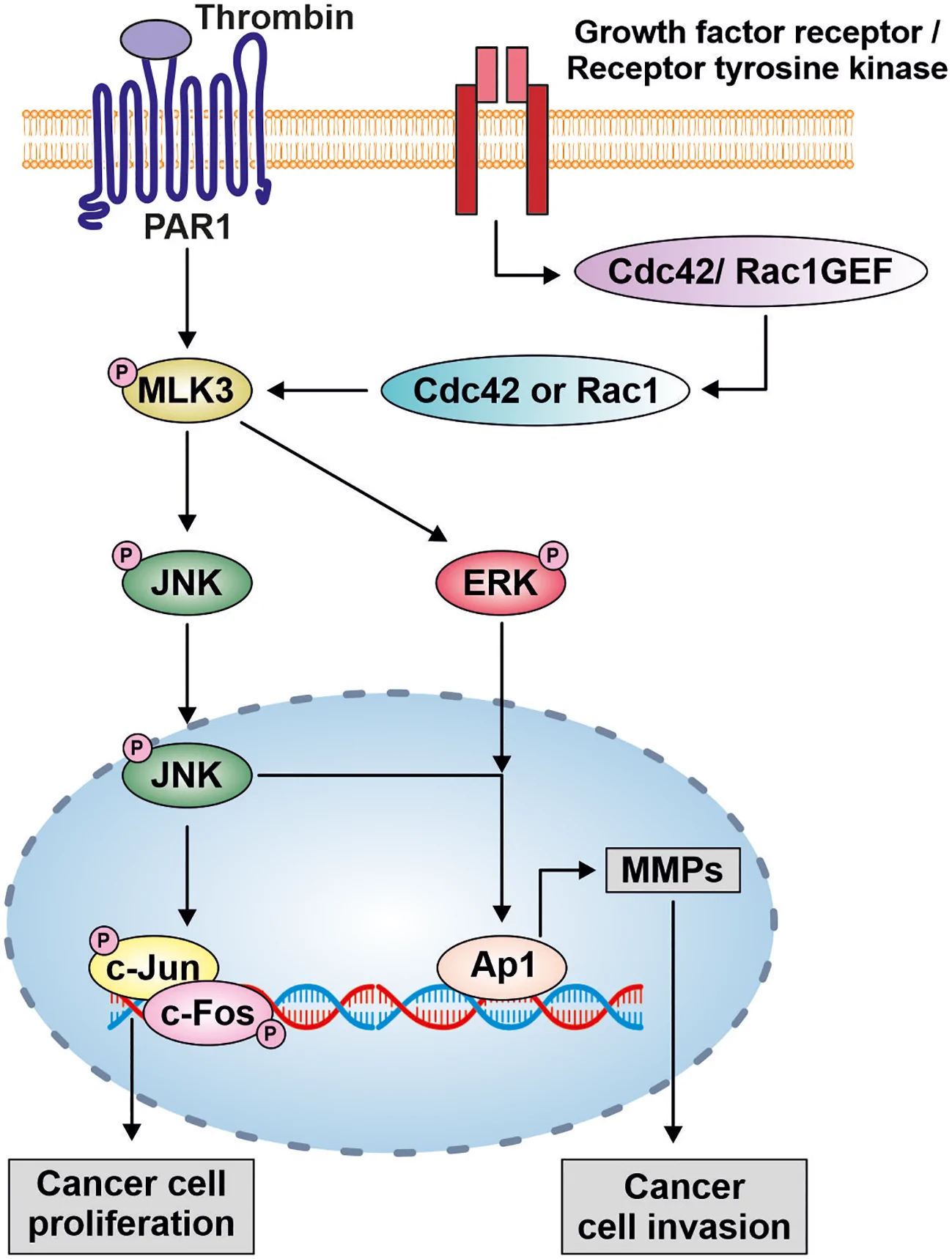
Thrombin-mediated activation of PAR1 stimulates MLK3, leading to activation of the JNK and ERK signaling pathways. Activated JNK enhances c-Jun/c-Fos-mediated AP-1 transcriptional activity, promoting expression of MMPs such as MMP-1 and MMP-9, which facilitate ECM degradation and tumor cell invasion. Cdc42 and Rac1, activated by guanine nucleotide exchange factors, function as upstream regulators of MLK3 and promote its membrane localization and activation, thereby contributing to cancer cell proliferation and metastatic progression in TNBC.

The PROTAC CEP1347-VHL-02 demonstrated rapid, selective MLK3 degradation via VHL in TNBC models (97). This approach may represent a potentially more complete suppression of MLK3 activity than kinase inhibition alone. MLK3 crosstalk with NF- κB and PI3K/AKT pathways raises the hypothesis that MLK3 inhibition may influence tumor immune evasion and synergize with ICIs (95). MLK3 expression and activity may serve as predictive biomarkers for treatment responsiveness, supporting a precision medicine approach. All current MLK3 inhibitor data in TNBC are preclinical as no clinical evidence exists in patients.

#### 5.4.3 FGFR inhibitors, Anti VEGFR strategies, and NTRK fusions

TNBC cell lines with H+FGFR1 or FGFR2 amplifications exhibit increased sensitivity to FGFR inhibitors like PD173074 and dovitinib in preclinical models (55), providing a biomarker defined subset hypothesis. Apatinib has demonstrated significant antitumor effects in TNBC by inhibiting VEGFR signalling. And treatment with apatinib and camrelizumab is found to effectively improve objective response rates in patients with mTNBC (55). A case report by Lv et al. described anti-tumour activity with low-dose apatinib in advanced TNBC (98). In addition, the prospective single arm phase II MA-BC-II-026 trial evaluated camrelizumab + apatinib and chemotherapy as neoadjuvant therapy in TNBC and reported improved ORR (99). While these preliminary results are encouraging, the absence of a randomized comparator arm limits conclusions about the independent contribution of apatinib to the observed benefit. Neurotrophic tropomyosin receptor kinase (NTRK) gene fusions are rare in breast cancer (<1% of cases). However, entrectinib and larotrectinib have received FDA approval for the treatment of NTRK fusion-positive solid tumours irrespective of histology (69). This supports a tissue-agnostic precision oncology opportunity for a small but defined TNBC fraction. MAPK inhibitors including trametinib combined with chemotherapy reduced dormant cancer cell fractions in preclinical models (46), but clinical application faces substantial challenges.

### 5.5 Notch pathway inhibitors

#### 5.5.1 Molecular pathogenesis and pathway dysregulation in TNBC

The Notch signaling cascade is crucial for cell fate determination, growth, and homeostasis (53, 100). This signaling includes four Notch receptors (Notch-1, 2, 3, and 4) and five ligands (Jagged-1, Jagged-2, Delta-like 1, Delta-like 3, and Delta-like 4). It is activated when transmembrane ligands bind to Notch receptors on the cell surface (53). This binding initiates proteolytic cleavage of Notch by a PSEN-1-dependent γ-secretase complex, freeing the Notch intracellular domain (NICD). The NICD then translocates to the nucleus, where it forms a heterodimer with the transcription factor CSL (suppressor of hairless) to activate target genes such as HES1, HEY2, MYC, CCND1, HES4, and NRAR (**Figure 7**). These target genes are crucial for the proliferation, differentiation, and apoptosis of BCSCs (53, 100). Notably, Miao et al. demonstrated that NOTCH1 activation compensates for BRCA1 deficiency and promotes TNBC formation via activation of the ATR-CHK1 signaling pathway. This mechanism restores S/G2 and G2/M cell cycle checkpoints function and protects BRCA1 deficient cells from cytotoxic stress (22). These findings establish a direct mechanistic link between Notch signaling and DNA repair pathway activation. This interaction may contribute to tumor formation and resistance to genotoxic therapy in BRCA1 deficient TNBC, and creating a rationale for PARP inhibitor and Notch inhibitors co-administration (22).

**Figure 7:**
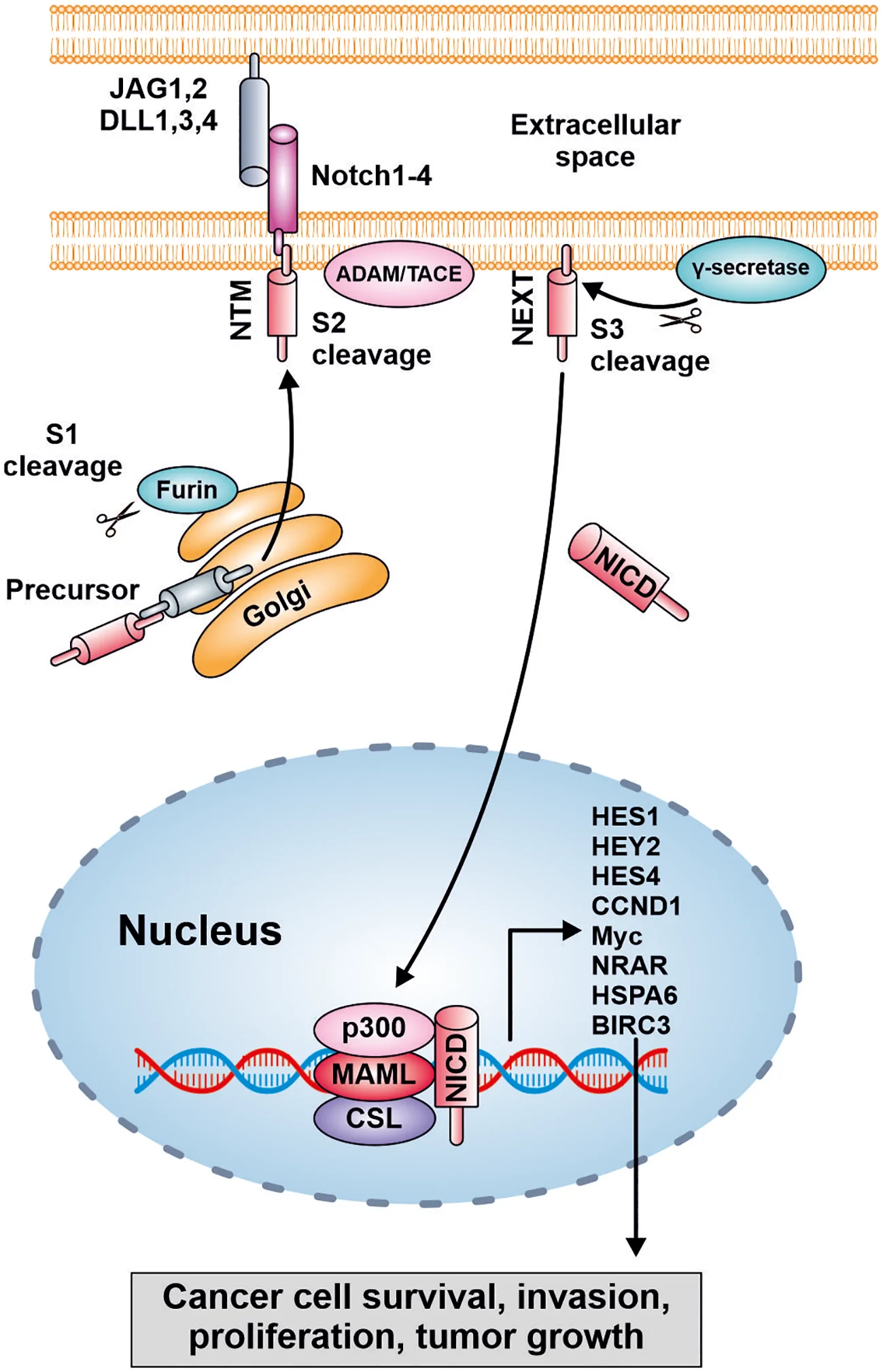
The Notch signaling pathway is initiated by interaction of Notch receptors (Notch1-4) with jagged or Delta-like ligands on adjacent cells. Ligand binding induces sequential proteolytic cleavages by ADAM/TACE and γ-secretase, releasing the Notch intracellular domain (NICD). NICD translocate to the nucleus, where it forms a transcriptional complex with CSL, MAML, and p300 to activate target genes including HES, HEY, CCND1, and MYC. Activation of these genes promotes TNBC cell survival, proliferation, invasion, and tumor progression.

The distinct roles of individual Notch receptors complicate therapeutic targeting. Notch-3 and Notch-4 have been linked to tumor initiation and proliferation, whereas Notch-2 might serve as a protective factor in TNBC cell lines (53, 55). Zhang et al. reported that Notch3 is overexpressed in TNBC and significantly co-expressed with EGFR, contributing to resistance to targeted therapies. In addition, Notch3 was shown to promote osteolytic bone metastasis, highlighting its clinical relevance beyond primary tumor progression (18). This receptor-specific heterogeneity raises a critical therapeutic challenge. Pan-Notch inhibition may non-selectively abrogate protective Notch2 signaling or produce excessive gastrointestinal toxicity due to Notch inhibition in intestinal crypts. This toxicity emerged as a dose limiting factor in early clinical trials of γ-secretase inhibitors (GSIs) (18). Targeting Notch1 with monoclonal antibodies reduces HES and HEY-L family gene expression in MDA-MB-231 cells (53), demonstrating that receptor-selective approaches are feasible.

#### 5.5.2 GSI: Clinical evidence and toxicity limitations

GSIs RO4929097 and MK-0752, have emerged as potential therapeutic agents. These inhibitors block the cleavage of Notch receptors, activation of downstream signaling (101, 102). Reduced taxol sensitivity has been found to occur due to overexpression of the catalytic subunit PSEN-1 which significantly increases γ-secretase activity. This leads to the accumulation of NICD in the nucleus and in conjunction with pregnane X receptor, it promotes the transcription of downstream drug resistance genes (103). Sardesai et al. conducted a phase I study of oral RO4929097 combined with neoadjuvant paclitaxel and carboplatin in TNBC. The regimen was tolerated as the recommended doses but without definitive efficacy data from this phase I study (104). Similarly, Schott et al. evaluated GSI with docetaxel in human across preclinical and clinical breast cancer models. Preclinical findings showed that GSIs could sensitize breast tumor- initiating cells to docetaxel in preclinical models. However, clinical translation was constrained by gastrointestinal toxicity, particularly diarrhea and intestinal goblet cell metaplasia resulting from on-target Notch inhibition in the gut (105). These clinical data identify toxicity as the primary translational barrier to GSI development. Toxicity at therapeutically relevant doses, which has limited the clinical development of this drug class despite compelling preclinical rationale.

#### 5.5.3 Novel delivery system and ligand specific strategies

The solid lipid nanoparticle delivery system targeting GSIs to TNBC cells via DR-5 receptors showed enhanced antitumor activity compared to free drug. Similarly, the anti-DR-5 ADC (Zaptuzumab) demonstrates greater therapeutic efficacy and safety than unmodified Zaptuzumab in preclinical models (100, 103). Anti-DLL4 monoclonal antibody inhibited breast tumor growth and suppressed BCSC-associated anti-apoptotic genes (HSPA6, BIRC3) through the Notch pathway (53, 55, 100). Combining anti-DLL4 antibodies with paclitaxel reduced BCSC frequency and delayed tumor relapse in paclitaxel resistant TNBCs (100). Notch signaling is upregulated in dormant cancer cell populations, with increased expression of jagged1, jagged2, delta-like 1 and 4 in the dormant extracellular matrix niche (46). In this context, Notch activation altered gene expression in an integrin-independent manner, suggesting that ECM composition influences cellular signaling beyond direct integrin engagement.

#### 5.5.4 Novel notch modulators and combination strategies

CB-103, a pan-Notch transcription complex inhibitor, combined with fulvestrant produced tumour regression in the ESR1-Y537S resistant model (106). In contrast, the combination showed limited benefit in ESR1 wild type tumours (106). These findings suggest that CB-103 may have greater therapeutic value in overcoming hormone therapy resistance. Sulindac sulfide and α-PD-1 significantly reduced tumour growth in TNBC compared to either treatment alone, demonstrating its efficacy as a γ-secretase modulator and in conjunction with an ICI (107). RNA sequencing identified 131 differentially expressed genes in tumours treated with sulindac sulfide, and further Ingenuity Pathways Analysis highlighted immunologically relevant pathways involving PKCθ, OX40, CTLA4, and PD-L1/PD-1, as well as pathways associated with CSC maintenance, including PI3K/AKT, Wnt, and ERK5 (107). Wang et al. further demonstrated that IL-6 blockade enhances the anti-tumour effects of GSAs in Notch3 expressing breast cancer. These findings provide a rationale for combining IL-6 inhibitors, such as tocilizumab, with Notch targeted therapies. Despite this, the combination strategy has not yet been evaluated in clinical trials in TNBC (102). The EPAC inhibitor Ny0123 activated Notch 1 signalling to promote endothelial growth arrest and reduce VEGFR2 expression, suggesting an anti-angiogenic mechanism partly via Notch (108). These multi-pathway interactions illustrate the biological plausibility of combining Notch inhibitors with immunotherapy and anti-angiogenic agents. However, they also underscore the complexity of predicting clinical outcomes from such combinations.

## 6 Importance of immunotherapies and immune response

ICIs exert their antitumor effects by blocking inhibitory immune checkpoint pathways that suppress T-cell activation and enable tumor immune evasion. ICIs target immune regulatory molecules such as CTLA-4, PD-1, and PD-L1, thereby restoring and enhancing antitumor immune responses (48, 49, 109, 110). Unlike other breast cancer subtypes, TNBC demonstrates limited responsiveness to hormone-based therapies (e.g., tamoxifen and aromatase inhibitors) and HER2-targeted treatments because of the absence of hormone receptor and HER2 expression. TNBC is characterized by a more aggressive clinical course, higher metastatic potential, and poorer prognosis than other breast cancer subtypes and occurs more frequently in younger women (48, 69). Despite this biological rationale, the clinical evidence supporting immunotherapy in TNBC remains heterogeneous. Regulatory approvals are limited to specific biomarker-defined populations, response rates remain modest in unselected cohorts, and several large trials have reported conflicting or negative results. A critical appraisal of the evidence is therefore essential to distinguish practice-changing findings from preliminary or inconclusive data. The TORCHLIGHT and FUTURESUPER trials further emphasize the importance of molecular subtype stratification, as immune-subtype TNBC appears to derive greater therapeutic benefit (55).

### 6.1 PD-1/PD-L1 inhibitors

#### 6.1.1 Biological rationale and biomarker landscape

Programmed death receptor ligand (PD-L1) along with its receptor PD-1 are exploited by cancer cells to evade immune surveillance (**Figure 8**). PD-L1 expression is significantly higher in TNBC (~19%) compared to the other breast cancer subtypes (~6.3%) and ~24% of TNBC cases are classified as immune subtypes with heightened ICI sensitivity (55). Beyond PD-L1, additional biomarkers have emerged as potential predictors of therapeutic response and prognosis. For instance, the TNBC-DX risk score 14-gene immunoglobulin-related immune profile has been associated with long-term clinical outcomes and pathological complete response in TNBC patients receiving neoadjuvant chemotherapy (111). However, PD-L1 expression is not uniformly assessed across trials. The SP142 assay used in IMpassion130 measures immune cell staining, whereas the 22C3 assay used in KEYNOTE trials measures combined positive score (CPS). These methodological differences limit the reliability of cross-trial comparisons (55). This assay heterogeneity is a fundamental methodological limitation that complicates the interpretation of PD-L1 as a predictive biomarker. The FDA recommends a dosing regimen of 840 mg IV infusion of atezolizumab over 60 minutes, followed by 100 mg/m² paclitaxel for a 28-day cycle for patients with advanced or mTNBC (53).

**Figure 8:**
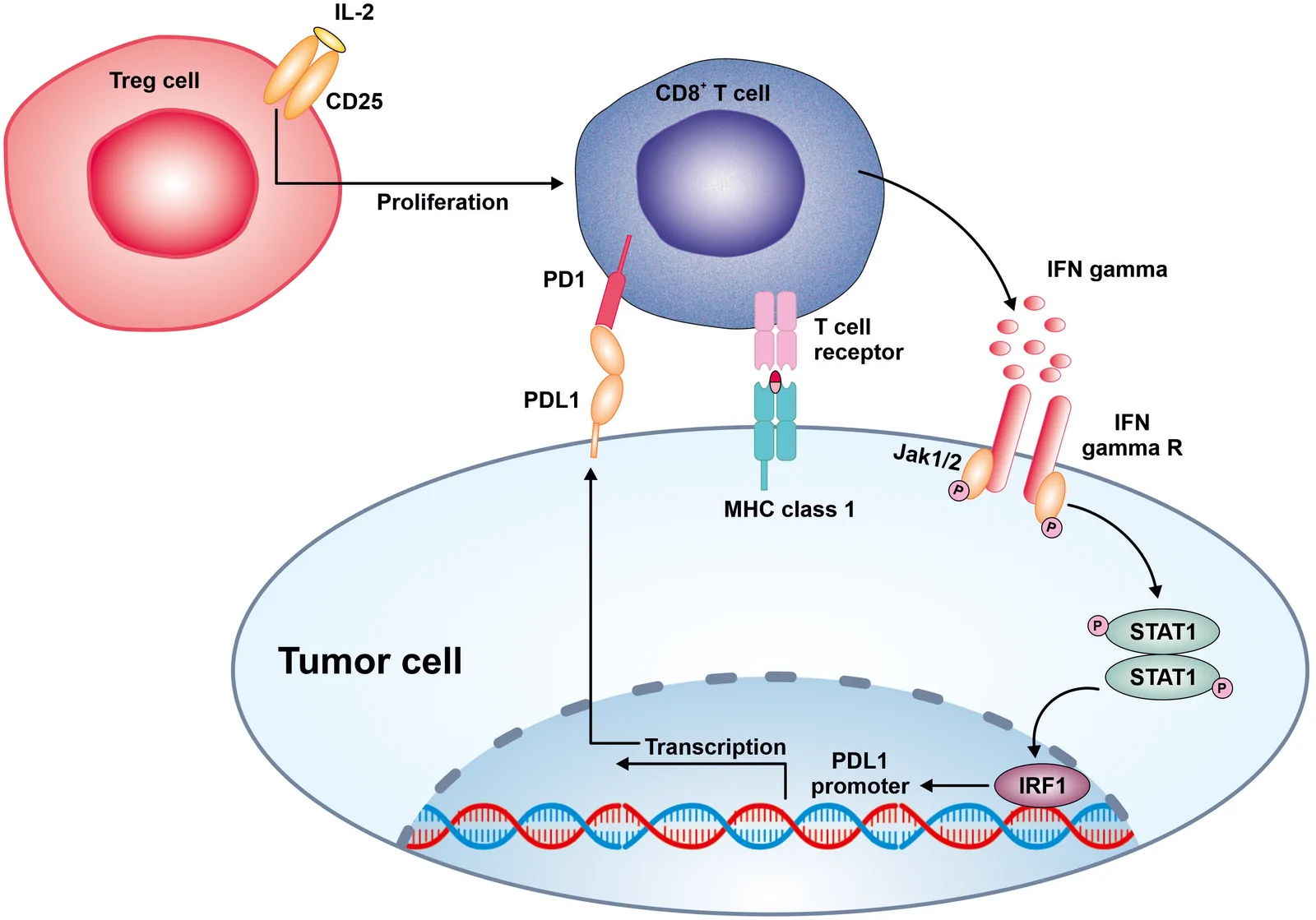
PD-L1 expressed on TNBC cells binds to PD-1 receptors on T cells, suppressing T-cell activation and enabling immune evasion. Blockade of the PD-1/PD-L1 axis with monoclonal antibodies such as nivolumab and pembrolizumab restores anti-tumor immune responses. IFN-γ/JAK/STAT signaling further upregulates PD-L1 expression, establishing a negative feedback mechanisms that limits T-cell-mediated cytotoxicity. High infiltration of regulatory T cells (Tregs) in TNBC contributes to immunosuppression, and CD25-targeted depletion of Tregs may enhance the therapeutic efficacy of PD-1/PD-L1 inhibitors and IL-12-based immunotherapy.

#### 6.1.2 ICIs in TNBC: regulatory status and clinical evidence

Atezolizumab (IMpassion130 vs IMpassion131): The IMpassion130 trial initially supported FDA accelerated approval of atezolizumab combined with nab-paclitaxel in PD-L1 positive mTNBC, reporting an ORR of 39% (112). However, this approval was subsequently withdrawn following the contradictory results of the IMpassion131 trial. In this study, atezolizumab combined with paclitaxel (solvent-based) failed to improve overall survival or PFS in the same population (55). The divergent outcomes of these two trials, which differed primarily in the taxane backbone, highlight the influence of chemotherapy selection on immunotherapy efficacy. These differences may reflect distinct immunomodulatory effects on the TME. This critical observation is often underemphasised in descriptive reviews but is central to understanding why atezolizumab no longer holds an approved indication in this setting. Similarly, IMpassion031 reported improved pCR rates with neoadjuvant atezolizumab + nab-paclitaxel (113). However, longer-term EFS data remain awaited, limiting firm conclusions about survival benefit (113).

Pembrolizumab (KEYNOTE-522): Pembrolizumab has the most robust approval in TNBC, supported by the KEYNOTE-522 phase III trial. The addition of pembrolizumab to neoadjuvant chemotherapy increased pCR rates by 7.5% and improved EFS from 79.4% to 86.6% (55, 110). Critically, the EFS benefit was observed regardless of PD-L1 status, which distinguishes pembrolizumab from atezolizumab and has important implications for patient selection. This was the basis for FDA approval in 2021 for high-risk early-stage TNBC (114). However, 44% of pembrolizumab recipients experienced immune-related adverse events (irAEs), and the absolute EFS gain must be weighed against this toxicity burden. This consideration is relevant for patients who would have achieved pCR with chemotherapy alone, who may derive limited additional benefit while bearing the full toxicity risk (55, 114). The KEYNOTE-355 trial demonstrated that pembrolizumab combined with chemotherapy improved PFS and overall survival in patients with PD-L1-positive metastatic TNBC, supporting its role in biomarker-selected advanced disease (55).

#### 6.1.3 Investigational agents and conflicting evidence

Durvalumab: Durvalumab demonstrated improved pCR rates in the GeparNuevo neoadjuvant trial, but the SAFIRO2-BREAST IMMUNO trial found that it did not improve PFS or overall survival in general breast cancer. Importantly, an exploratory subgroup analysis of SAFIRO2 reported prolonged overall survival specifically in TNBC patients. However, this observation should be interpreted with caution given its *post-hoc*, hypothesis-generating nature and the risk of type I error in underpowered subgroup analyses (55). These results should not be treated as equivalent in evidence quality to primary endpoint data from prospective trials. Durvalumab is also being investigated alongside neoantigen vaccines in several early-phase trials (109).

Avelumab: Avelumab monotherapy in the phase Ib JAVELIN trial showed a 5.2% ORR in unselected TNBC, rising to 22.2% in PD-L1-positive cases (49, 109). These ORR figures, derived from single-arm phase I data, represent hypothesis-generating preliminary evidence rather than practice-changing results. Without randomized comparison arms, it is not possible to attribute response rates to avelumab specifically or to distinguish them from natural disease fluctuation. Furthermore, avelumab has not proceeded to phase III evaluation in TNBC, and its clinical role remains undefined (49).

Pembrolizumab Monotherapy (KEYNOTE-086 and KEYNOTE-119): These two trials present an instructive contrast. KEYNOTE-086 reported a 21.4% ORR with pembrolizumab monotherapy in PD-L1-positive previously treated TNBC. While the randomized KEYNOTE-119 phase III trial, representing much higher-quality evidence, found no significant improvement in overall survival vs. chemotherapy in a similar population (49, 55). A divergence between single-arm ORR data and randomized survival outcomes has been consistently observed in TNBC immunotherapy. This finding highlights the limitations of using ORR as a surrogate endpoint for overall survival benefit. KEYNOTE-119 effectively constrains the single-arm enthusiasm generated by KEYNOTE-086, yet both findings are often cited without this critical hierarchy.

#### 6.1.4 Combination strategies: mechanistic rationale and limitations

Beyond direct PD-1/PD-L1 blockade, several rational combination strategies have been pursued. Preclinical evidence linking the Ras/MAPK pathway to suppression of antigen presentation and PD-L1 regulation has motivated the combination of MEK inhibitors with PD-1/PD-L1 blockade (10). Although enhanced efficacy has been demonstrated in murine syngeneic models, translation to human TNBC has been challenging. This is largely attributable to overlapping toxicities and the immunosuppressive effects of certain MEK inhibitors at therapeutically relevant doses. These limitations are often underrepresented in reviews that primarily emphasize preclinical findings (10). Similarly, IRX-2, a multi-cytokine formulation designed to prime the TME, has demonstrated increases in TILs and PD-L1 expression in early-stage TNBC, with a follow-up phase II trial ongoing (NCT04373031) (110). Bempegaldesleukin, a pegylated IL-2 variant targeting CD122, increases TILs and T cell clonality when combined with anti-PD-1 therapy. However, IL-2-based therapies carry substantial risks of vascular leak syndrome and systemic toxicity, and no phase III data in TNBC are yet available. Targeting CD25 to selectively reduce regulatory T cells (Tregs) while preserving IL-2 signalling represents a mechanistically appealing approach but remains at the preclinical or early clinical stage (110).

#### 6.1.5 Cancer vaccines combined with ICIs: early-phase evidence

Several clinical trials are currently evaluating vaccine–ICI combinations in TNBC. These include pembrolizumab combined with PVX-410 (NCT03362060), p53-targeting vaccines (NCT02432963), WT1 vaccines (NCT03761914), and atezolizumab in combination with neoantigen vaccines (NCT03289962) (49, 109). These trials are predominantly phase I/II and primarily designed to assess safety and immunogenicity rather than clinical efficacy endpoints. Despite encouraging immunogenicity, the clinical potential of therapeutic cancer vaccines remains uncertain, as vaccine-induced immune responses have historically translated poorly into objective tumor regression in solid tumors (110). Nelipepimut-S combined with trastuzumab showed clinical benefit specifically in the TNBC subgroup of a phase II HER2-low breast cancer trial. Despite this was a subgroup analysis of a small trial, and the findings require prospective validation in a TNBC-specific cohort before clinical adoption (110). Oncolytic virotherapy with T-VEC combined with atezolizumab yielded objective responses in only 2 of 10 patients in a phase I HER2-negative breast cancer trial post-neoadjuvant chemotherapy. These findings highlight the limited clinical activity and small sample sizes characteristic of early-phase oncoviral trials (110).

### 6.2 CLTA-4 inhibitors

#### 6.2.1 Mechanistic role and approved context

CTLA-4 functions as a competitive inhibitor of CD28-mediated T cell co-stimulation by binding CD80/CD86 with higher affinity, and by inhibiting downstream PI3K, GRB2, and filamin signaling. Ipilimumab, by blocking CTLA-4, restores CD28 engagement and facilitates T cell activation (109). The ipilimumab-nivolumab combination has received FDA approval in melanoma, renal cell carcinoma, and microsatellite instability-high colorectal cancer, based on randomized phase III data demonstrating OS benefit. However, no CTLA-4 inhibitor currently holds an approved indication in TNBC, and all evidence in this subtype remains preclinical or derived from early-phase trials (31). This fundamental distinction between approved indications in other tumor types and investigational status in TNBC is critical and must not be conflated.

#### 6.2.2 Preclinical and early clinical evidence in TNBC

In preclinical TNBC models, the combination of anti-CTLA-4 antibodies with a MUC1 mRNA nano-vaccine induced significant apoptosis in 4T1 cells and suppressed tumor growth in mice (10). In the mTNBC murine setting, combining a CTLA-4 inhibitor with the microtubule-targeting agent DZ-2384 improved survival (10). These findings were derived from murine models and should not be directly extrapolated to clinical outcomes in humans. The immunological architecture of syngeneic murine tumors differs substantially from human TNBC, particularly in MHC composition, Treg abundance, and PD-L1 dynamics. Therefore, while these data support mechanistic rationale, they must be regarded as hypothesis-generating rather than evidence of clinical efficacy (10, 55).

In clinical studies, tremelimumab combined with exemestane in HR-positive breast cancer demonstrated increased proliferating CD4+ and CD8+ T cells. Similarly, ipilimumab combined with cryoablation enhanced T cell responses in breast cancer patients (109). Both observations stem from small, early-phase studies with immunological endpoints rather than survival outcomes. The absence of randomised phase III evidence for CTLA-4 inhibition in TNBC indicates that this strategy remains investigational. In addition, the considerable irAE burden of dual checkpoint blockade (particularly grade 3–4 colitis, hepatitis, and endocrinopathies) representing a significant barrier to its broad clinical adoption, especially in a patient population that may be receiving concurrent cytotoxic chemotherapy (109).

### 6.3 HER2 (CAR) T-cell therapy

#### 6.3.1 Target antigens: status and evidence grade

A critical evaluation of CAR T-cell therapy in TNBC must emphasize that virtually all evidence in this setting remains preclinical. Unlike hematological malignancies where CD19-directed CAR T-cells have received FDA approval based on phase II data, no CAR T-cell therapy has yet achieved regulatory approval for TNBC or any solid tumor (10). Solid tumors present unique challenges, including physical barriers to T cell trafficking, the immunosuppressive TME, antigen heterogeneity, and CAR T-cell exhaustion. These factors likely contribute to the substantial gap between promising preclinical results and clinical translation (10, 55).

Mesothelin: Mesothelin is overexpressed in TNBC and minimally expressed in normal tissues, making it an attractive CAR T target (10). However, mesothelin-directed CAR T-cell trials in mesothelioma and ovarian cancer have demonstrated limited clinical activity despite robust preclinical signals. These results are encouraging but should be interpreted cautiously the general challenge of solid tumor immunotherapy. No mesothelin-directed CAR T data specific to TNBC from randomized trials currently exist (10).

AXL: A receptor tyrosine kinase belonging to the TAM family (Tyros, AXL, MER), has been shown to be highly expressed on the surface of MDA-MB-231 cells. Studies have successfully constructed AXL-CAR-T cells, which exhibited significant cytotoxic effects against MDA-MB-231 cells *in vitro* and effectively inhibited the growth of subcutaneous xenografts in mouse models (10). Xenograft models using immunodeficient mice cannot reproduce the immunologically intact human TME, meaning that efficacy in these systems may not predict human clinical responses. The immunosuppressive signaling capacity of AXL itself, through its role in efferocytosis and TGF-β induction, also poses a potential mechanisms of resistance to CAR T-cell therapy that remains to be addressed.

MUC1, ROR1 and EGFRvIII: CAR T-cell approaches targeting MUC1 and ROR1 are in early development and have shown antitumor activity in TNBC models, with ROR1-directed CAR T cells exhibiting a favourable preclinical safety profile (55). CAR natural killer (NK) cells targeting EGFRvIII and tissue factor have demonstrated promising efficacy in preclinical studies (55). Notably, MUC1 is expressed on many normal epithelial tissues, raising concerns about on-target off-tumor toxicity that have not been fully characterized in TNBC-specific models. The field of CAR NK cells is even earlier in development than CAR T cells, and all cited evidence is preclinical. These approaches should be presented as emerging strategies under active investigation rather than as near-term therapeutic options.

**Supplementary Table 1** shows all the targeted and immune-based therapeutic strategies in TNBC.

## 7 Emerging strategies

The application of aptamers in order to accelerate body’s self-defenses against different types of cancers is an emerging strategy for managing TNBC (115). Other targeted methods involve discovering biomarkers to forecast resistance to particular chemotherapeutic drugs and altering plans for therapy accordingly. Furthermore, research is being conducted on immune-checkpoint inhibitors, anti-angiogenic medications, inhibitors of HSP 90 and histone deacetylase, and targeted therapies based on signaling pathways (69, 116). In an attempt to develop novel treatment options, research has also concentrated on understanding tumor heterogeneity, transcriptome dysregulation, and the immunological microenvironment of TNBC.

### 7.1 Novel drug delivery systems

To treat breast cancer which has been triple negative, for which there are no appropriate treatment choices, novel ways to deliver drugs are being researched (TNBC). Among these, nanocarrier-based systems, including extracellular vesicles (EVs), polymeric nanoparticles, liposomes, and solid lipid nanoparticles have been studied as potential vehicles for delivering chemotherapeutic agents to TNBC cells with greater precision and reduced off-target toxicity (117–119). It is important to note that the majority of evidence supporting these approaches currently derives from *in vitro* cell line studies and *in vivo* animal model. Clinical translation remains at an early stage, and significant challenges related to scalability, reproducibility, and safety profiling must still be addressed before routine clinical implementation can be achieved (120).

Nanocarriers can be functionalized with targeting ligands and loaded with chemotherapeutic agents to improve selective uptake by TNBC cells. Their attractiveness as a delivery platform lies in properties such as tunable drug loading, controlled release mechanisms, and reduced systemic cytotoxicity compared to conventional chemotherapy (118, 120). Notably, liposomal doxorubicin formulations such as Doxil^®^ represent the most clinically advanced nanocarrier application, having received FDA approval for use in breast cancer. However, these are not TNBC-specific and do not reflect the targeted nanocarrier strategies currently under preclinical investigation (120).

At the preclinical level, EV-based drug delivery systems have attracted particular interest. A systematic review of 17 studies published between 2020 and 2024 evaluated the therapeutic potential of EV-based delivery platforms in TNBC and reported encouraging *in vitro* and *in vivo* findings. These included effective drug loading, marked anti-proliferative activity, and substantial tumor accumulation in cell line models (119). However, the review also emphasized that clinical translation remains a future objective and requires further validation (117). Among the preclinical models investigated, macrophage-derived EVs loaded with paclitaxel (PTX) and doxorubicin (Dox) have demonstrated anticancer activity in TNBC cell lines. In these studies, EVs functioned as delivery vehicles that enabled more precise intracellular transport of therapeutic agents. This targeted delivery was associated with inhibition of tumor cell growth *in vitro* (117). However, these findings have not yet been replicated in clinical trial settings, and translating such results from cell line and mouse models to human patients remains a significant and recognized challenge in the field, as conventional TNBC cell line models often fail to capture the full molecular and genetic heterogeneity of human tumors (120).

In summary, nanocarrier- and EV-based drug delivery strategies show promising preclinical potential in TNBC, but standardization, further *in vivo* validation, and early-phase clinical studies are required before clinical application.

### 7.2 Artificial intelligence in TNBC diagnosis, prognosis, and treatment decision support

The integration of Big Data encompassing clinical histories, treatment outcomes, recurrence rates, and prognostic factors holds considerable promise for transforming oncologists’ approaches to TNBC (121). AI-based methodologies, including machine learning, deep learning, radiomics, and explainable AI, enable the integration and analysis of multidimensional datasets encompassing genomic, transcriptomic, histopathological, radiological, and clinical information to identify clinically relevant patterns beyond conventional analytical approaches (122, 123). Through these analytical capabilities, AI is increasingly being integrated into TNBC research and clinical management as a computational and clinical decision-support methodology rather than a therapeutic intervention. AI approaches support diagnostic classification, molecular subtyping, biomarker discovery, prognosis prediction, treatment response assessment, and optimization of clinical decision-making. By facilitating multidimensional data interpretation, AI contributes to precision oncology and supports individualized patient management in TNBC (121, 124).

Beyond diagnostics, AI has demonstrated utility in therapeutic planning and clinical decision support. The DESIREE (Decision Support and Information Management System for Breast Cancer) platform, developed by Séroussi et al. (125), integrates explainable AI and case-based reasoning to support treatment decision-making in breast cancer. Validation by practicing oncologists positions DESIREE among the more clinically advanced AI systems in this field (125). Predictive models have further shown capacity to forecast response to neoadjuvant chemotherapy and ICIs, enabling patient stratification for immunotherapy and potentially reducing unnecessary treatment-related toxicity. Such predictive capabilities may improve patient stratification and support more individualized therapeutic approaches in TNBC. Furthermore, AI-driven drug discovery platforms are increasingly being applied to identify novel molecular targets and optimize therapeutic combinations via large-scale analysis of omics datasets. Emerging evidence suggests that AI-guided computational approaches can accelerate the discovery of candidate drug pairs and enhance treatment selection strategies in TNBC (126, 127). At the preclinical level, the machine learning algorithm idTRAX has been applied to predict TNBC cell line sensitivity to targeted agents, including AKT and FGFR2 inhibitors, though these findings are currently confined to *in vitro* models and await clinical validation (128) (**Figure 9**).

**Figure 9:**
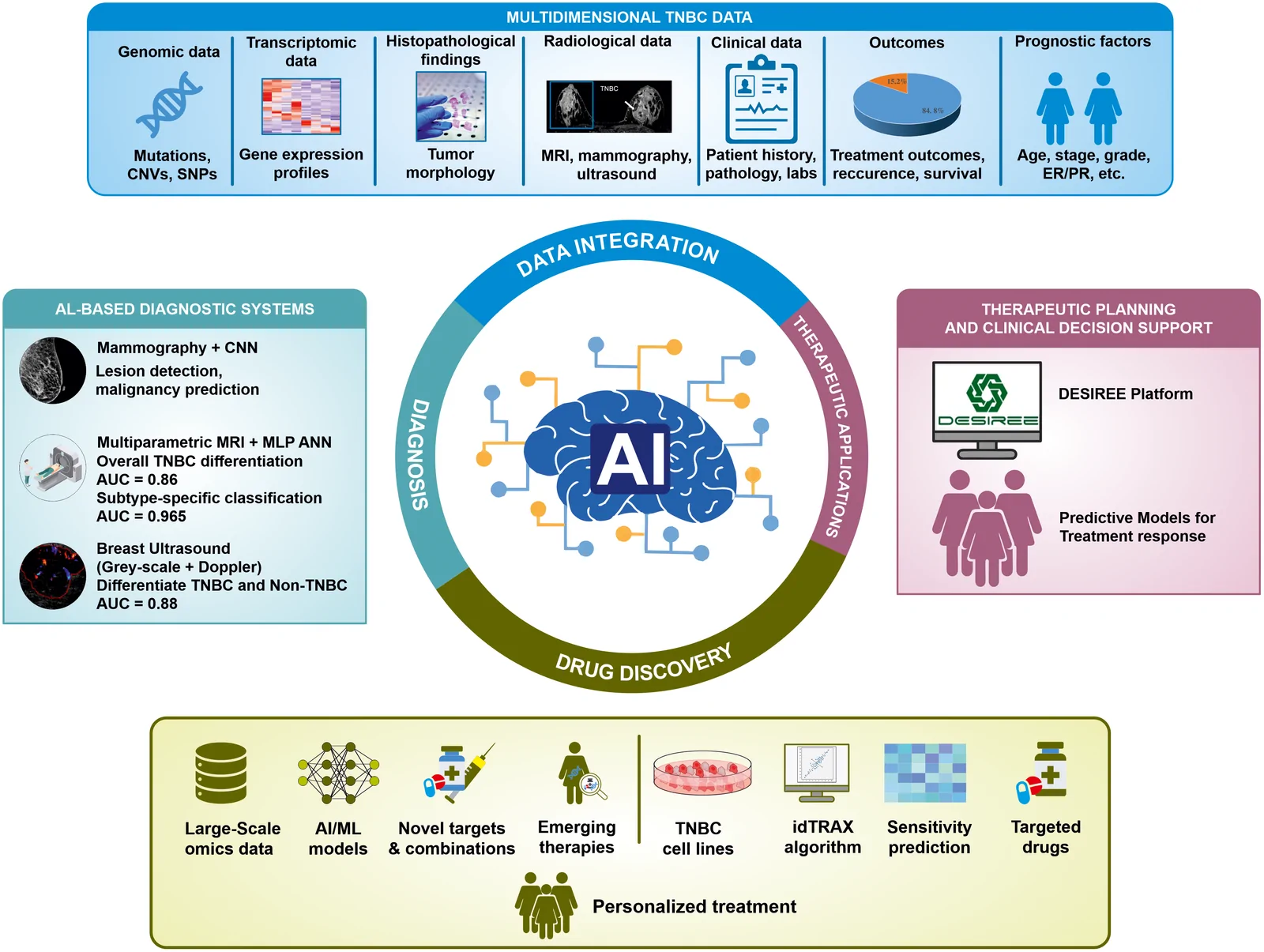
AI-driven precision oncology framework for TNBC illustrating integration of multidimensional data for diagnosis, treatment prediction, clinical decision support, drug discovery, and personalized therapy.

Collectively, AI constitutes a rapidly evolving and multifaceted tool for improving TNBC diagnosis, therapeutic prediction, and drug discovery. However, the majority of current applications remain at research or early validation stages. Rigorous prospective trials, multicenter studies, and integration into standardized clinical workflows are essential conditions for the broad clinical translation of AI-based approaches in oncological practice (121, 129).

### 7.3 ADCs in cancer therapy

ADCs represent a promising therapeutic strategy by merging the potent cytotoxic effects of chemotherapy with the targeted specificity of antibodies.

Nectin-4 (also known as PVRL4) is a cell adhesion molecule that has been identified as a potential biomarker and therapeutic target in TNBC (130). M-Rabet et al. developed an ADC, named N41mab-vcMMAE, and consists of a human anti-necin-4 monoclonal antibody linked to monomethyl auristatin E (MMAE), a potent cytotoxic agent. It demonstrated high affinity and specificity for nectin-4, leading to effective internalization in nectin-4-expressing cell lines. In TNBC cases, membranous high nectin-4 protein expression was found in approximately 62% of the cases (131).

Trophoblast cell-surface antigen 2 (Trop-2) is a transmembrane glycoprotein encoded by the TACSTD2 gene, and it is found to be overexpressed in TNBC cases (132). Sacituzumab govitecan, also known as Trodelvy, is an ADC that targets Trop-2. It is a novel ADC that combines the humanized RS7 anti-TROP-2 monoclonal antibody with SN-38, the active metabolite of irinotecan (130). The IMMU-132–01, ASCENT, TROPiCS-02, NeoSTAR and SASCIA trial have investigated the sacituzumab govitecan potential as neoadjuvant therapy for early-stage TNBC (55, 130). These trials highlight sacituzumab govitecan’s superiority over traditional chemotherapy in improving PFS and overall survival for patients with recurrent or refractory TNBC and for heavily pretreated, endocrine-resistant HR+/HER2− subtypes (55). Several ongoing trials such as Saci-IO (NCT04468061; Phase II), Morpheus TNBC (NCT03424005; Phase Ib/II), InCITe (NCT03971409; Phase II), Phase I/II with Talazoparib, ASCENT-03 and ASCENT-04 (Phase III) continue to investigate acituzumab govitecan in various settings (130). Datopotamab deruxtecan is a TROP-2-directed ADC that consists of a humanized anti-TROP-2 IgG1 monoclonal antibody linked to a potent topoisomerase I inhibitor (Deruxtecan) via a cleavable tetrapeptide-based linker (55, 130). The drug-to-antibody ratio is approximately 4:1 (130). Deruxtecan is approximately 10 times more potent than SN-38, the active metabolite of irinotecan used in sacituzumab govitecan. Considering the potential of topoisomerase I inhibitors to enhance the efficacy of ICIs, a phase Ib/II trial (BEGONIA) is assessing Datopotamab-deruxtecan in combination with durvalumab for first-line mTNBC. And in 27 patients, the confirmed overall response rate was found to be 74% (130). DS-8201a is an HER2-targeted ADC, made up of an anti-HER2 antibody linked to a derivative of the topoisomerase I inhibitor DX-8951. Phase II/III trials have shown that DS-8201a provides significant tumor inhibitory activity in HER2-positive metastatic breast cancer and has been approved (55).

## 8 Conclusion

Recent breakthroughs in the field of oncology have sparked considerable enthusiasm in the quest for effective treatments for TNBC. A variety of therapy approaches are being developed based on the particular molecules and signaling pathways that are expressed in TNBC. The exploration of targeted therapies has notably gained attraction, with an emphasis on unravelling the complex molecular landscape of the disease. In essence, these therapeutic strategies exhibits inherent potentials and well as pitfalls while being administered in isolation. Therefore, the confluence of diverse targeted therapies emerges as a superior strategy to amplify therapeutic efficacy and extend benefits to a broader spectrum of TNBC patients. Moreover, imperative is the need for extensive and nuanced investigations to pinpoint novel and effective therapeutic targets in TNBC. It is expected that in the imminent future, individuals with TNBC would benefit from a broader selection of personalized treatment choices, ultimately resulting in enhanced results.

## Glossary

ADCs: Antibody-Drug Conjugates
AR: Androgen Receptor
BCSC: Breast Cancer Stem Cell
BRCA: BReast CAncer gene
CAFs: Cancer Associate Fibroblasts
CAR: Chimeric Antigen Receptor
CCI: Charlson Comorbidity Index
CDK: Cyclin-dependent kinase
CLTA4: Cytotoxic T-Lymphocyte-Associated Protein 4
COL: Collagen
CSC: Cancer Stem Cell
CTLs: Cytotoxic T Lymphocytes
DDR: Discoidin Domain Receptors
DNMT: DNA methyltransferase
ECM: Extra Cellular Matrix
EFS: Event-Free Survival
EGFR: Epidermal Growth Factor Receptor
EMT: Epithelial-Mesenchymal Transition
ER: Estrogen Receptor
GSI: γ-secretase inhibitors
HER2: Human Epidermal Growth Factor Receptor 2
Hh pathway: Hedgehog Pathway
MDSC: Myeloid Derived Suppress Cell
MET: Mesenchymal-Epithelial Transition factor
MLK3: Mixed-lineage kinase 3
MMPs: Matrix Metalloproteinases
mTOR: Mechanistic Target of Rapamycin
MYC: Myelocytomatosis
NICD: Notch intracellular domain
NTRK: Neurotrophic Tyrosine Receptor Kinase
PARP: Poly ADP-Ribose Polymerase
pCR: Pathological Complete Response
PD-L1: Programmed Death Receptor Ligand
PFS: Progression Free Survival
PI3K: Phosphoinositide 3 Kinase
PIK3CA: Phosphatidylinositol 4, 5-Bisphosphate 3-Kinase Catalytic Subunit Alpha
PR: Progesterone Receptor
SIK2: Salt-Inducible Kinase 2
TAMs: Tumor-Associated Macrophages
TBK1: TANK-binding kinase-1
TIL: Tumor-Infiltrating Lymphocyte
TME: Tumor Microenvironment
TNBC: Triple-Negative Breast Cancer
Trop2: Trophoblast cell-surface antigen 2
VEGFR: Vascular Endothelial Growth Factor Receptor
