# Supplementary material for: Recent advances in targeted therapies and immunotherapies for triple-negative breast cancer
Source: Front Oncol. 2026 Sep 28;16:1804620. doi: 10.3389/fonc.2026.1804620 (PMC13656090; doi:10.3389/fonc.2026.1804620)
Supplement: Supplementary file 1 [file Table1.docx]

Supplementary Table 1. Overview of precision oncology strategies in TNBC, summarizing targeted and immunotherapeutic agents, molecular targets, clinical development status, efficacy outcomes, safety profiles, and key clinical limitations.

| **Drug (Trial)** | **Phase & Study design** | **Clinical population** | **Target** | **Biomarker Eligibility** | **Status** | **Treatment line** | **Primary Endpoint** | **Key outcomes** | **Safety** | **Clinical significance** | **Limitations** | **Ref.** |
| --- | --- | --- | --- | --- | --- | --- | --- | --- | --- | --- | --- | --- |
|  | *P*ARP inhibitors | | | | | | | | | | | |
| Olaparib (OlympiA) | Phase III, randomized, double-blind, placebo-controlled | Patients with high-risk HER2-negative early breast cancer carrying gBRCA1/2 mutations, including TNBC (n=1836) | PARP1/2 | gBRCA1/2 mutation | FDA-approved | Adjuvant (early-stage) | iDFS | Improved iDFS, DDFS, OS | Anemia, fatigue, nausea | Standard adjuvant therapy for gBRCA-mutated TNBC | Limited to gBRCA cancer patients | [1-4] |
| Niraparib (BRAVO) | Phase III, randomized, open-label | Patients with gBRCA mutated advanced or metastatic HER2-negative breast cancer, including TNBC (n=306) | PARP1/2 | gBRCA1/2 mutation | Investigational | Metastatic | PFS | Modest PFS improvement; ongoing evaluation in combination regimens | Nausea, fatigue, thrombocytopenia | Potential immunotherapy combination strategy | No FDA approval for TNBC; limited efficacy as monotherapy | [5-7] |
| Rucaparib (Phase II study) | Phase II, open-label, single-arm | Patients with metastatic or advanced BRCA1/2-mutated breast cancer, including TNBC (n=204) | PARP1/2 | BRCA1/2 mutation | Investigational | Previously treated metastatic | ORR | Limited ORR; possible TME modulation | Fatigue, nausea, anemia | Supports immunomodulatory potential of PARP inhibition | Limited clinical efficacy; no regulatory approval in breast cancer | [8, 9] |
| Talazoparib (EMBRACA) | Phase III, open-label, randomized | Patients with gBRCA-mutated HER2-negative advanced breast cancer, including TNBC (n=431) | PARP1/2 | gBRCA1/2 mutation | FDA-approved | Metastatic | PFS | Significantly improved PFS and quality of life | Anemia, neutropenia | Preferred PARP inhibitor for metastatic gBRCA-mutated disease | Restricted to gBRCA-mutated patients | [1, 10-13] |
| Talazoparib (NEOTALA) | Phase II, single-arm, open-label | Patients with gBRCA-mutated early-stage HER2-negative breast cancer, including TNBC | PARP1/2 | gBRCA1/2 mutation | Investigational | Neoadjuvant | pCR | Encouraging pCR with single-agent talazoparib | Anemia, neutropenia | Demonstrates feasibility of chemotherapy-free neoadjuvant PARP inhibition | Single-arm design without comparator |  |
| Veliparib (BrighTNess) | Phase III, randomized, double-blind, placebo-controlled | Patients with stage II-III TNBC receiving neoadjuvant carboplatin-based chemotherapy (n=634) | PARP1/2 | None required | Investigational | Neoadjuvant | pCR | Improved pCR; no additional EFS benefit | Myelosuppression | Supports platinum-based neoadjuvant therapy rather than veliparib-specific benefit | No long-term survival advantage; weak PARP trapping activity | [14-17] |
|  | Immune Checkpoint Inhibitors- Immunotherapy | | | | | | | | | | | |
| Pembrolizumab (KEYNOTE-522) | Phase III, randomized, double-blind, placebo-controlled | Patients with high-risk early-stage TNBC receiving neoadjuvant chemotherapy followed by adjuvant therapy (n=1174) | PD-1 | None required | FDA-approved | Neoadjuvant → Adjuvant | pCR, EFS | Improved pCR and EFS | irAEs endocrinopathies, pneumonitis, hepatitis | Established immunotherapy option | Long-term immune-related toxicities require monitoring; increased treatment complexity and cost. | [18-20] |
| Atezolizumab (IMpassion130) | Phase III, randomized, double-blind, placebo-controlled | Patients with unresectable locally advanced or metastatic TNBC receiving nab-paclitaxel (n=902) | PD-L1 | PD-L1-positive immune cells (SP142 IC ≥1%) | FDA indication withdrawn | First-line metastatic | PFS, OS | Improved PFS and ORR; OS benefit observed in PD-L1-positive subgroup | irAEs: hepatic enzyme elevation | Demonstrated importance of biomarker-selected immunotherapy | Clinical benefit largely restricted to PD-L1-positive tumors; regulatory indication later withdrawn after conflicting confirmatory results. | [1, 21-25] |
| Ipilimumab (Single-arm Phase II Study) | Phase II, single-arm, open-label | Patients with early-stage and metastatic TNBC receiving CTLA-4 blockade (n=34) | CTLA-4 | None established | Investigational | Neoadjuvant / Metastatic | pCR | Promising pCR signals and immune activation | Colitis, diarrhea, hepatitis | Supports dual checkpoint blockade | Small sample size; lack of validated predictive biomarkers; substantial immune-related toxicity. | [26-29] |
| Tremelimumab (Pilot Study) | Phase I/II, open-label pilot study | Patients with locally advanced or metastatic breast cancer including TNBC subgroup (n=18) | CTLA-4 | None established | Investigational | Metastatic | ORR | Modest ORR benefit in TNBC subgroup | Grade 3-4 colitis, diarrhea, hepatitis | Encourages CTLA-4 combinations | Small cohort; high irAEs like colitis and diarrhea | [27, 30-32] |
| Mesothelin CAR-T Cell Therapy (Phase I Dose-Escalation Study) | Phase I, dose-escalation, open-label | Patients with advanced mesothelin-positive solid tumors including TNBC (n=109) | Mesothelin | Mesothelin-positive tumors | Investigational | Advanced / Metastatic | Safety | Feasible; preliminary anti-tumor activity | Cytokine release syndrome (CRS) risk; neurotoxicity (ICANS) | Promising adoptive cellular immunotherapy strategy | Complex manufacturing process; high cost; risk of CRS and neurotoxicity. | [26, 33-35] |
| PI3K / AKT / mTOR Pathway Inhibitors | | | | | | | | | | | | |
| Alpelisib + Nab-paclitaxel (Phase I/II Study) | Phase I/II, open-label, non-randomized | Patients with metastatic HER2-negative breast cancer harboring PIK3CA alterations, including TNBC subgroup (n=12) | PI3Kα (PIK3CA) | PIK3CA mutation | Investigational | Metastatic | ORR | Promising ORR and clinical benefit rate | Hyperglycemia, neutropenia | Supports biomarker-guided PI3K-targeted therapy | Small TNBC subgroup; non-randomized design; resistance frequently develops during treatment. | [36-39] |
| Everolimus (Phase II Study) | Phase II, open-label | Patients with metastatic TNBC receiving mTOR-targeted therapy (n=25) | mTOR | None established | Investigational | Metastatic | CBR | Modest activity; improved clinical benefit in combination regimens | Stomatitis, fatigue, hyperglycemia, pneumonitis | Potential combination partner for targeted therapy | Small sample size; limited single-agent efficacy; potential immunosuppressive effects. | [1, 40-42] |
| Capivasertib (PAKT) | Phase II, randomized, double-blind, placebo-controlled | Patients with first-line metastatic TNBC harboring PI3K/AKT/PTEN pathway alterations (n=140) | AKT | PIK3CA/AKT1/PTEN alterations | Investigational | First-line metastatic | OS | Improved OS and PFS | Hyperglycemia, rash (capivasertib class effects); diarrhea; manageable with dose modification | Supports biomarker-guided AKT inhibition | Not specifically approved for TNBC; efficacy greatest in biomarker-selected populations. | [43, 44] |
| Ipatasertib (LOTUS) | Phase II, randomized, double-blind, placebo-controlled | Patients with metastatic TNBC receiving paclitaxel-based therapy (n=124) | AKT | PTEN-low and PI3K/AKT pathway-altered tumors | Investigational | First-line metastatic | PFS | Improved PFS, particularly in PTEN-low tumors | Diarrhea, nausea, fatigue | Supports AKT-targeted therapy in biomarker-selected TNBC | Benefit mainly observed in PTEN-low tumors; phase III validation remains limited. | [39, 40, 45] |
|  | Standard Chemotherapy | | | | | | | | | | | |
| Paclitaxel (Multiple Phase III Studies) | Phase III, randomized controlled trials | Patients with early-stage and metastatic TNBC receiving taxane-based chemotherapy (n=902) | β-Tubulin | None required | FDA-approved | Neoadjuvant, Adjuvant, and Metastatic | PFS | Effective standard chemotherapy backbone | Peripheral neuropathy, neutropenia | Core TNBC chemotherapy backbone | Dose-limiting neuropathy and hematologic toxicity may require treatment modification. | [15, 22, 46-48] |
| Doxorubicin (Translational Biomarker Study) | Retrospective observational translational study | Patients with early-stage TNBC receiving anthracycline-based neoadjuvant therapy (n=18) | Topoisomerase II / DNA damage pathway | CREB3L1 expression | FDA-approved | Neoadjuvant | pCR | Improved pCR when incorporated into combination regimens | Cumulative cardiotoxicity: cardiomyopathy (dose-dependent | Established neoadjuvant backbone | Small cohort; retrospective design; cumulative cardiotoxicity limits long-term use. | [48-51] |
|  | EGFR Inhibitors | | | | | | | | | | | |
| Cetuximab (Randomized Phase II Study) | Phase II, randomized | Patients with metastatic TNBC receiving cetuximab-based therapy (n=173) | EGFR | None established | Investigational | First- or second-line metastatic | ORR | Improved ORR with modest PFS benefit | Acneiform rash, gastrointestinal toxicity | Supports EGFR-targeted combination approaches | Limited efficacy as monotherapy; EGFR expression alone poorly predicts response. | [52-56] |
| Panitumumab (Multicenter Phase II Study) | Phase II, multicenter, single-arm | Patients with stage II–III TNBC receiving neoadjuvant EGFR-targeted therapy (n=60) | EGFR | None established | Investigational | Neoadjuvant | pCR | Improved pCR; predictive gene signatures identified | Severe acneiform rash, hypomagnesemia, gastrointestinal toxicity | Supports biomarker discovery for EGFR-targeted therapy | No control arm; substantial dermatologic toxicity limits tolerability. | [57, 58] |
| Erlotinib (Phase I Study) | Phase I, open-label | Patients with previously treated metastatic TNBC receiving erlotinib-based therapy (n=8) | EGFR | None established | Investigational | Previously treated metastatic | Safety | Limited antitumor activity; manageable safety profile | Lymphopenia, rash | Highlights need for biomarker-guided EGFR inhibition | Very small cohort; low response rates in unselected TNBC populations. | [59-61] |
|  | OTHER KINASES INHIBITORS | | | | | | | | | | | |
| Ganitumab (I-SPY2) | Phase II, adaptive randomized clinical trial | Patients with stage II–III TNBC receiving neoadjuvant IGF1R-targeted therapy (n=106) | IGF1R | IGF1R pathway activation | Investigational | Neoadjuvant | pCR | Limited efficacy with modest improvement in pCR rates | Hematologic, gastrointestinal, and metabolic AEs. | Provided important biological insight into IGF1R-directed therapy in TNBC | Limited clinical benefit; metabolic toxicity profile; lack of validated predictive biomarkers for patient selection. | [62, 63] |
| Dovitinib (Phase II Study) | Phase II, non-randomized, open-label | Patients with metastatic TNBC, including FGFR-amplified subgroups (n=81) | FGFR | FGFR amplification | Investigational | Metastatic | ORR | Limited overall efficacy; responses enriched in FGFR-amplified tumors | Nausea, vomiting, diarrhea, fatigue | Highlights the importance of biomarker-selected FGFR inhibition in TNBC | Low response rates; heterogeneous study population; absence of confirmatory phase III data. | [1, 64] |
| Apatinib (Multicenter Phase II Study) | II Phase II, multicenter, single-arm | Patients with advanced or metastatic TNBC receiving VEGFR-targeted therapy (n=38) | VEGFR | None established | Investigational | Refractory Metastatic | PFS | Modest ORR, PFS, and OS benefit | Hypertension; hand-foot syndrome; proteinuria; fatigue | Potential option for refractory TNBC | Single-arm design without randomized comparator; lack of predictive biomarkers limits patient selection. | [65-67] |
|  | NOTCH Pathway inhibitor | | | | | | | | | | | |
| RO4929097 (Phase I Study) | Phase I, open-label | Patients with early-stage or metastatic TNBC receiving γ-secretase inhibitor-based therapy (n=14) | γ-secretase (NOTCH pathway) | None established | Investigational | Neoadjuvant / Metastatic | Safety | Demonstrated feasibility of NOTCH pathway inhibition in combination regimens | Nausea, diarrhea , GI toxicity | Provides proof-of-concept for NOTCH-targeted therapy in TNBC | Small sample size; significant gastrointestinal toxicity limited dosing and long-term treatment feasibility. | [68-71] |
| CB-103 (Phase I/II Study) | Phase I/II, dose-escalation, open-label | Patients with advanced or metastatic solid tumors, including TNBC (sample size not reported) | NOTCH transcription complex | NOTCH pathway activation | Investigational | Advanced / Metastatic | Safety and tolerability | Preliminary antitumor activity observed with evidence of pathway inhibition | Fatigue, nausea, vomiting | Supports direct targeting of NOTCH signaling in TNBC and other solid tumors | Early-phase study with limited TNBC-specific data; low response rates and heterogeneous study population. | [72, 73] |
|  | Antibody-Drug Conjugates (ADCs) | | | | | | | | | | | |
| Sacituzumab Govitecan (ASCENT) | Phase III, randomized, open-label | Patients with previously treated metastatic TNBC (n=529) | TROP-2 | None required | FDA-approved | Previously treated metastatic | PFS | Improved PFS, OS, and ORR | Neutropenia, leukopenia, diarrhea, anemia | Standard for metastatic TNBC | High treatment cost and limited accessibility in some healthcare settings. | [74-76] |
|  | Androgen Receptor (AR) Inhibitors | | | | | | | | | | | |
| Enzalutamide (MDV3100-11) | Phase II, single-arm, open-label | Patients with androgen receptor-positive TNBC enrolled in a phase II study (n=118) | Androgen Receptor (AR) | AR-positive TNBC | Investigational | Metastatic | CBR | Clinical benefit in AR-positive TNBC | Fatigue, hot flashes | Validates AR as a therapeutic target in molecularly selected TNBC | Applicable only to AR-positive disease; modest overall response rates. | [77, 78] |

| Bicalutamide (AR Antagonist) | II | 424 | Androgen Receptor | At investigational stage | Metastatic /Advance | CBR at (6 months); PFS | Bicalutamide monotherapy showed modest activity (CBR ~19%) in AR+ mTNBC (LAR subtype); confirmed AR as a therapeutic target in this subtype | Common AEs neutropenia, leukopenia, acneiform rash, fatigue, and diarrhea, consistent with the expected toxicity profile of EGFR inhibition combined with chemotherapy. | Demonstrated promising neoadjuvant efficacy of EGFR-targeted therapy in TNBC, particularly in biologically defined subgroups likely to benefit from panitumumab-based treatment. | No approved indication for TNBC; limited to AR-positive (~10–20%) TNBC subgroup | [3, 79, 80] |
| --- | --- | --- | --- | --- | --- | --- | --- | --- | --- | --- | --- |

iDFS-Invasive Disease-Free; PFS- Progression-Free Survival; OS-Overall Survival; mOS-Median Overall Survival; ORR- Objective response rate; pCR- pathological complete response; EFS- event-free survival; DLTs- dose-limiting toxicity; irAEs-Immue related adverse events; CR-Complete Response; PR-Partial Response ;CBR-Clinical Benefit Rate; AC- Adriamycin: Cyclophosphamide; DCR- disease control rate; RP2D- recommended Phase II dose; gBRCA-germline BRCA; Ref.-Reference

1. Zhu S, Wu Y, Song B, Yi M, Yan Y, Mei Q, Wu K. Recent advances in targeted strategies for triple-negative breast cancer. J Hematol Oncol (2023) 16:100. doi: 10.1186/s13045-023-01497-3
2. Tutt ANJ, Garber JE, Kaufman B, Viale G, Fumagalli D, Rastogi P, Gelber RD, de Azambuja E, Fielding A, Balmaña J, et al. Adjuvant Olaparib for Patients with BRCA1- or BRCA2-Mutated Breast Cancer. N Engl J Med (2021) 384:2394–2405. doi: 10.1056/NEJMoa2105215
3. Ferrari P, Scatena C, Ghilli M, Bargagna I, Lorenzini G, Nicolini A. Molecular Mechanisms, Biomarkers and Emerging Therapies for Chemotherapy Resistant TNBC. Int J Mol Sci (2022) 23:1665. doi: 10.3390/ijms23031665
4. Arora S, Balasubramaniam S, Zhang H, Berman T, Narayan P, Suzman D, Bloomquist E, Tang S, Gong Y, Sridhara R, et al. FDA Approval Summary: Olaparib Monotherapy or in Combination with Bevacizumab for the Maintenance Treatment of Patients with Advanced Ovarian Cancer. Oncologist (2021) 26:e164–e172. doi: 10.1002/onco.13551
5. Luo L, Keyomarsi K. PARP inhibitors as single agents and in combination therapy: the most promising treatment strategies in clinical trials for BRCA-mutant ovarian and triple-negative breast cancers. Expert Opin Investig Drugs (2022) 31:607–631. doi: 10.1080/13543784.2022.2067527
6. Subhan MA, Parveen F, Shah H, Yalamarty SSK, Ataide JA, Torchilin VP. Recent Advances with Precision Medicine Treatment for Breast Cancer including Triple-Negative Sub-Type. Cancers (Basel) (2023) 15:2204. doi: 10.3390/cancers15082204.
7. Chauhan K, Kumari P, Devi D, Thakur S, Thakur S, Bisht K, Bala M. Niraparib in breast cancer: evidence-based perspective on translational progress. Breast Cancer (2026) 33:569–581. doi: 10.1007/s12282-026-01856-1
8. Halder R, Bhamidipati N, Kiss A, Aldhahri W, Ehsani S, Segar JM, Foster N, Chalasani P. Change in tumor microenvironment and PD-L1 expression with rucaparib treatment in triple-negative breast cancer. JCO (2024) 42:e12569–e12569. doi: 10.1200/JCO.2024.42.16_suppl.e12569
9. Swisher EM, Lin KK, Oza AM, Scott CL, Giordano H, Sun J, Konecny GE, Coleman RL, Tinker AV, O’Malley DM, et al. Rucaparib in relapsed, platinum-sensitive high-grade ovarian carcinoma (ARIEL2 Part 1): an international, multicentre, open-label, phase 2 trial. The Lancet Oncology (2017) 18:75–87. doi: 10.1016/S1470-2045(16)30559-9
10. Won K-A, Spruck C. Triple‑negative breast cancer therapy: Current and future perspectives (Review). Int J Oncol (2020) 57:1245–1261. doi: 10.3892/ijo.2020.5135
11. Litton JK, Beck JT, Jones JM, Andersen J, Blum JL, Mina LA, Brig R, Danso M, Yuan Y, Abbattista A, et al. Neoadjuvant Talazoparib in Patients With Germline BRCA1/2 Mutation-Positive, Early-Stage Triple-Negative Breast Cancer: Results of a Phase II Study. Oncologist (2023) 28:845–855. doi: 10.1093/oncolo/oyad139
12. Liu X, Ge Z, Yang F, Contreras A, Lee S, White JB, Lu Y, Labrie M, Arun BK, Moulder SL, et al. Identification of biomarkers of response to preoperative talazoparib monotherapy in treatment naïve gBRCA+ breast cancers. NPJ Breast Cancer (2022) 8:64. doi: 10.1038/s41523-022-00427-9
13. US Food and Drug Administration. FDA approves talazoparib for gBRCAm HER2-negative locally advanced or metastatic breast cancer. Silver Spring (MD): US Food and Drug Administration (2018 Sep).
14. Geyer CE, Sikov WM, Huober J, Rugo HS, Wolmark N, O’Shaughnessy J, Maag D, Untch M, Golshan M, Lorenzo JP, et al. Long-term efficacy and safety of addition of carboplatin with or without veliparib to standard neoadjuvant chemotherapy in triple-negative breast cancer: 4-year follow-up data from BrighTNess, a randomized phase III trial. Ann Oncol (2022) 33:384–394. doi: 10.1016/j.annonc.2022.01.009
15. Loibl S, O’Shaughnessy J, Untch M, Sikov WM, Rugo HS, McKee MD, Huober J, Golshan M, von Minckwitz G, Maag D, et al. Addition of the PARP inhibitor veliparib plus carboplatin or carboplatin alone to standard neoadjuvant chemotherapy in triple-negative breast cancer (BrighTNess): a randomised, phase 3 trial. Lancet Oncol (2018) 19:497–509. doi: 10.1016/S1470-2045(18)30111-6
16. Stringer-Reasor EM, May JE, Olariu E, Caterinicchia V, Li Y, Chen D, Della Manna DL, Rocque GB, Vaklavas C, Falkson CI, et al. An open-label, pilot study of veliparib and lapatinib in patients with metastatic, triple-negative breast cancer. Breast Cancer Res (2021) 23:30. doi: 10.1186/s13058-021-01408-9
17. Afifa A, Saddique S, Saeed H, Inam H, Saddique RA, Rehman HM, Hussain N. Harnessing genomic profiling for precision breast cancer therapies: Advances in HER2-targeted treatments, PARP inhibitors for BRCA mutations, and insights from clinical trials. Biomedical Research and Therapy (2026) 13:8234–8256. doi: 10.15419/v1dhqx66	(139)
18. Pauls M, Chia S, LeVasseur N. Current and New Novel Combination Treatments for Metastatic Triple-Negative Breast Cancer. Curr Oncol (2022) 29:4748–4767. doi: 10.3390/curroncol29070377
19. McKeon J, Daniels E, Halstead A, Machado C, Potter S, Strawson-Smith T, Okines A, Mcintosh S, Tovey H, Robinson T, et al. UK real-world evidence on pembrolizumab and neoadjuvant chemotherapy for early-stage triple-negative breast cancer. ESMO Real World Data Digit Oncol (2026) 11:100664. doi: 10.1016/j.esmorw.2025.100664
20. Kwapisz D. Pembrolizumab and atezolizumab in triple-negative breast cancer. Cancer Immunol Immunother (2021) 70:607–617. doi: 10.1007/s00262-020-02736-z
21. Mittendorf EA, Zhang H, Barrios CH, Saji S, Jung KH, Hegg R, Koehler A, Sohn J, Iwata H, Telli ML, et al. Neoadjuvant atezolizumab in combination with sequential nab-paclitaxel and anthracycline-based chemotherapy versus placebo and chemotherapy in patients with early-stage triple-negative breast cancer (IMpassion031): a randomised, double-blind, phase 3 trial. Lancet (2020) 396:1090–1100. doi: 10.1016/S0140-6736(20)31953-X
22. Schmid P, Adams S, Rugo HS, Schneeweiss A, Barrios CH, Iwata H, Diéras V, Hegg R, Im S-A, Shaw Wright G, et al. Atezolizumab and Nab-Paclitaxel in Advanced Triple-Negative Breast Cancer. N Engl J Med (2018) 379:2108–2121. doi: 10.1056/NEJMoa1809615
23. Emens LA, Cruz C, Eder JP, Braiteh F, Chung C, Tolaney SM, Kuter I, Nanda R, Cassier PA, Delord J-P, et al. Long-term Clinical Outcomes and Biomarker Analyses of Atezolizumab Therapy for Patients With Metastatic Triple-Negative Breast Cancer: A Phase 1 Study. JAMA Oncol (2019) 5:74–82. doi: 10.1001/jamaoncol.2018.4224
24. Narayan P, Wahby S, Gao JJ, Amiri-Kordestani L, Ibrahim A, Bloomquist E, Tang S, Xu Y, Liu J, Fu W, et al. FDA Approval Summary: Atezolizumab Plus Paclitaxel Protein-bound for the Treatment of Patients with Advanced or Metastatic TNBC Whose Tumors Express PD-L1. Clin Cancer Res (2020) 26:2284–2289. doi: 10.1158/1078-0432.CCR-19-3545
25. Gianni L, Huang CS, Egle D, Bermejo B, Zamagni C, Thill M, Anton A, Zambelli S, Bianchini G, Russo S, et al. Pathologic complete response (pCR) to neoadjuvant treatment with or without atezolizumab in triple-negative, early high-risk and locally advanced breast cancer: NeoTRIP Michelangelo randomized study. Ann Oncol (2022) 33:534–543. doi: 10.1016/j.annonc.2022.02.004
26. Yin L, Duan J-J, Bian X-W, Yu S-C. Triple-negative breast cancer molecular subtyping and treatment progress. Breast Cancer Res (2020) 22:61. doi: 10.1186/s13058-020-01296-5
27. Gaynor N, Crown J, Collins DM. Immune checkpoint inhibitors: Key trials and an emerging role in breast cancer. Semin Cancer Biol (2022) 79:44–57. doi: 10.1016/j.semcancer.2020.06.016
28. Loi S, Francis PA, Zdenkowski N, Gebski V, Fox SB, White M, Kiely BE, Hui R, Redfern AD, Calvert R, et al. Phase II study of neoadjuvant ipilimumab and nivolumab in combination with paclitaxel following anthracycline-based chemotherapy in patients with treatment resistant stage III triple negative breast cancer (TNBC): BCT1702—Survival results. JCO (2024) 42:608–608. doi: 10.1200/JCO.2024.42.16_suppl.608
29. Wang X, Wang L, Liu Y. Current Status of Immune Checkpoint Inhibitors and Treatment Responsive Biomarkers for Triple-Negative Breast Cancer. Thorac Cancer (2025) 16:e70072. doi: 10.1111/1759-7714.70072
30. Santa-Maria CA, Kato T, Park J-H, Kiyotani K, Rademaker A, Shah AN, Gross L, Blanco LZ, Jain S, Flaum L, et al. A pilot study of durvalumab and tremelimumab and immunogenomic dynamics in metastatic breast cancer. Oncotarget (2018) 9:18985–18996. doi: 10.18632/oncotarget.24867
31. Hossain F, Majumder S, David J, Miele L. Precision Medicine and Triple-Negative Breast Cancer: Current Landscape and Future Directions. Cancers (Basel) (2021) 13:3739. doi: 10.3390/cancers13153739
32. Jiang D (Maria), Fyles A, Nguyen LT, Neel BG, Sacher A, Rottapel R, Wang BX, Ohashi PS, Sridhar SS. Phase I study of local radiation and tremelimumab in patients with inoperable locally recurrent or metastatic breast cancer. Oncotarget (2019) 10:2947–2958. doi: 10.18632/oncotarget.26893
33. Yang P, Cao X, Cai H, Feng P, Chen X, Zhu Y, Yang Y, An W, Yang Y, Jie J. The exosomes derived from CAR-T cell efficiently target mesothelin and reduce triple-negative breast cancer growth. Cell Immunol (2021) 360:104262. doi: 10.1016/j.cellimm.2020.104262
34. Tchou J, Wang L-C, Selven B, Zhang H, Conejo-Garcia J, Borghaei H, Kalos M, Vondeheide RH, Albelda SM, June CH, et al. Mesothelin, a novel immunotherapy target for triple negative breast cancer. Breast Cancer Res Treat (2012) 133:799–804. doi: 10.1007/s10549-012-2018-4
35. Parinyanitikul N, Blumenschein GR, Wu Y, Lei X, Chavez-MacGregor M, Smart M, Gonzalez-Angulo AM. Mesothelin expression and survival outcomes in triple receptor negative breast cancer. Clin Breast Cancer (2013) 13:10.1016/j.clbc.2013.05.001. doi: 10.1016/j.clbc.2013.05.001
36. Savas P, Lo LL, Luen SJ, Blackley EF, Callahan J, Moodie K, van Geelen CT, Ko Y-A, Weng C-F, Wein L, et al. Alpelisib Monotherapy for PI3K-Altered, Pretreated Advanced Breast Cancer: A Phase II Study. Cancer Discov (2022) 12:2058–2073. doi: 10.1158/2159-8290.cd-21-1696
37. File DM, Abdou Y, Force J, Moore DT, Anders CK, Reeder-Hayes K, Carey LA, Muss HB, Perou CM, Marcom PK, et al. A Phase I Trial of Alpelisib Combined With Capecitabine in Patients With HER2-Negative Metastatic Breast Cancer. Clin Breast Cancer (2024) 24:683–690. doi: 10.1016/j.clbc.2024.08.001
38. Sharma, P., Abramson, V. G., O'Dea, A., Nye, L., Mayer, I., Pathak, H. B., Hoffmann, M., Stecklein, S. R., Elia, M., Lewis, S., Scott, J., De Jong, J. A., Wang, Y. Y., Yoder, R., Schwensen, K., Finke, K., Heldstab, J., LaFaver, S., Williamson, S. K., Phadnis, M. A., … Godwin, A. K. (2021). Clinical and Biomarker Results from Phase I/II Study of PI3K Inhibitor Alpelisib plus Nab-paclitaxel in HER2-Negative Metastatic Breast Cancer. Clinical cancer research : an official journal of the American Association for Cancer Research, 27(14), 3896–3904. https://doi.org/10.1158/1078-0432.CCR-20-4879
39. Cocco S, Leone A, Roca MS, Lombardi R, Piezzo M, Caputo R, Ciardiello C, Costantini S, Bruzzese F, Sisalli MJ, et al. Inhibition of autophagy by chloroquine prevents resistance to PI3K/AKT inhibitors and potentiates their antitumor effect in combination with paclitaxel in triple negative breast cancer models. J Transl Med (2022) 20:290. doi: 10.1186/s12967-022-03462-z
40. Khan MA, Jain VK, Rizwanullah M, Ahmad J, Jain K. PI3K/AKT/mTOR pathway inhibitors in triple-negative breast cancer: a review on drug discovery and future challenges. Drug Discov Today (2019) 24:2181–2191. doi: 10.1016/j.drudis.2019.09.001
41. Singh J, Novik Y, Stein S, Volm M, Meyers M, Smith J, Omene C, Speyer J, Schneider R, Jhaveri K, et al. Phase 2 trial of everolimus and carboplatin combination in patients with triple negative metastatic breast cancer. Breast Cancer Res (2014) 16:R32. doi: 10.1186/bcr3634
42. Yi Z, Ma F. Biomarkers of Everolimus Sensitivity in Hormone Receptor-Positive Breast Cancer. J Breast Cancer (2017) 20:321–326. doi: 10.4048/jbc.2017.20.4.321
43. Schmid P, Abraham J, Chan S, Wheatley D, Brunt AM, Nemsadze G, Baird RD, Park YH, Hall PS, Perren T, et al. Capivasertib Plus Paclitaxel Versus Placebo Plus Paclitaxel As First-Line Therapy for Metastatic Triple-Negative Breast Cancer: The PAKT Trial. J Clin Oncol (2020) 38:423–433. doi: 10.1200/JCO.19.00368
44. Nierengarten MB. FDA approves capivasertib with fulvestrant for breast cancer. Cancer (2024) 130:835–836. doi: 10.1002/cncr.35238
45. Kim S-B, Dent R, Im S-A, Espié M, Blau S, Tan AR, Isakoff SJ, Oliveira M, Saura C, Wongchenko MJ, et al. Ipatasertib plus paclitaxel versus placebo plus paclitaxel as first-line therapy for metastatic triple-negative breast cancer (LOTUS): a multicentre, randomised, double-blind, placebo-controlled, phase 2 trial. Lancet Oncol (2017) 18:1360–1372. doi: 10.1016/S1470-2045(17)30450-3
46. Abouzeid HA, Kassem L, Liu X, Abuelhana A. Paclitaxel resistance in breast cancer: Current challenges and recent advanced therapeutic strategies. Cancer Treat Res Commun (2025) 43:100918. doi: 10.1016/j.ctarc.2025.100918
47. Calistri NL, Liby TA, Hu Z, Zhang H, Dane MA, Gross SM, Heiser LM. TNBC response to paclitaxel phenocopies interferon response which reveals cell cycle-associated resistance mechanisms. Sci Rep (2025) 15:4294. doi: 10.1038/s41598-024-82218-9
48. Mandapati A, Lukong KE. Triple negative breast cancer: approved treatment options and their mechanisms of action. J Cancer Res Clin Oncol (2023) 149:3701–3719. doi: 10.1007/s00432-022-04189-6
49. Wu L, Ding W, Wang X, Li X, Yang J. Interference KRT17 reverses doxorubicin resistance in triple-negative breast cancer cells by Wnt/β-catenin signaling pathway. Genes Genomics (2023) 45:1329–1338. doi: 10.1007/s13258-023-01437-y
50. Denard B, Jiang S, Peng Y, Ye J. CREB3L1 as a potential biomarker predicting response of triple negative breast cancer to doxorubicin-based chemotherapy. BMC Cancer (2018) 18:813. doi: 10.1186/s12885-018-4724-8
51. Zeichner SB, Terawaki H, Gogineni K. A Review of Systemic Treatment in Metastatic Triple-Negative Breast Cancer. Breast Cancer (Auckl) (2016) 10:25–36. doi: 10.4137/BCBCR.S32783
52. Cheung A, Chenoweth AM, Johansson A, Laddach R, Guppy N, Trendell J, Esapa B, Mavousian A, Navarro-Llinas B, Haider S, et al. Anti-EGFR Antibody-Drug Conjugate Carrying an Inhibitor Targeting CDK Restricts Triple-Negative Breast Cancer Growth. Clin Cancer Res (2024) 30:3298–3315. doi: 10.1158/1078-0432.CCR-23-3110
53. Medina MA, Oza G, Sharma A, Arriaga LG, Hernández Hernández JM, Rotello VM, Ramirez JT. Triple-Negative Breast Cancer: A Review of Conventional and Advanced Therapeutic Strategies. Int J Environ Res Public Health (2020) 17:2078. doi: 10.3390/ijerph17062078
54. Carey LA, Rugo HS, Marcom PK, Mayer EL, Esteva FJ, Ma CX, Liu MC, Storniolo AM, Rimawi MF, Forero-Torres A, et al. TBCRC 001: randomized phase II study of cetuximab in combination with carboplatin in stage IV triple-negative breast cancer. J Clin Oncol (2012) 30:2615–2623. doi: 10.1200/JCO.2010.34.5579
55. Barbieri L, Salvioni L, Banfi A, Garbujo S, Fiandra L, Baioni C, Giustra M, Morelli L, Frascotti G, Colombo M, et al. Dual-Targeting Strategy to Repurpose Cetuximab with HFn Nanoconjugates for Immunotherapy of Triple-Negative Breast Cancer. ACS Appl Mater Interfaces (2025) 17:33648–33663. doi: 10.1021/acsami.5c06626
56. Baselga J, Gómez P, Greil R, Braga S, Climent MA, Wardley AM, Kaufman B, Stemmer SM, Pêgo A, Chan A, et al. Randomized phase II study of the anti-epidermal growth factor receptor monoclonal antibody cetuximab with cisplatin versus cisplatin alone in patients with metastatic triple-negative breast cancer. J Clin Oncol (2013) 31:2586–2592. doi: 10.1200/JCO.2012.46.2408
57. Nabholtz JM, Abrial C, Mouret-Reynier MA, Dauplat MM, Weber B, Gligorov J, Forest AM, Tredan O, Vanlemmens L, Petit T, et al. Multicentric neoadjuvant phase II study of panitumumab combined with an anthracycline/taxane-based chemotherapy in operable triple-negative breast cancer: identification of biologically defined signatures predicting treatment impact. Ann Oncol (2014) 25:1570–1577. doi: 10.1093/annonc/mdu183
58. Yardley DA, Ward PJ, Daniel BR, Eakle JF, Lamar RE, Lane CM, Hainsworth JD. Panitumumab, Gemcitabine, and Carboplatin as Treatment for Women With Metastatic Triple-Negative Breast Cancer: A Sarah Cannon Research Institute Phase II Trial. Clin Breast Cancer (2016) 16:349–355. doi: 10.1016/j.clbc.2016.05.006
59. Fenn K, Maurer M, Lee SM, Crew KD, Trivedi MS, Accordino MK, Hershman DL, Kalinsky K. Phase 1 Study of Erlotinib and Metformin in Metastatic Triple-Negative Breast Cancer. Clin Breast Cancer (2020) 20:80–86. doi: 10.1016/j.clbc.2019.08.004
60. Layman RM, Ruppert AS, Lynn M, Mrozek E, Ramaswamy B, Lustberg MB, Wesolowski R, Ottman S, Carothers S, Bingman A, et al. Severe and prolonged lymphopenia observed in patients treated with bendamustine and erlotinib for metastatic triple negative breast cancer. Cancer Chemother Pharmacol (2013) 71:1183–1190. doi: 10.1007/s00280-013-2112-2
61. Ma Y, Fang Z, Zhang H, Qi Y, Mao Y, Zheng J. PDZK1 suppresses TNBC development and sensitizes TNBC cells to erlotinib via the EGFR pathway. Cell Death Dis (2024) 15:199. doi: 10.1038/s41419-024-06502-2
62. Yee D, Isaacs C, Wolf DM, Yau C, Haluska P, Giridhar KV, Forero-Torres A, Jo Chien A, Wallace AM, Pusztai L, et al. Ganitumab and metformin plus standard neoadjuvant therapy in stage 2/3 breast cancer. NPJ Breast Cancer (2021) 7:131. doi: 10.1038/s41523-021-00337-2
63. Godina C, Pollak MN, Jernström H. Targeting IGF-IR improves neoadjuvant chemotherapy efficacy in breast cancers with low IGFBP7 expression. npj Precis Onc (2024) 8:212. doi: 10.1038/s41698-024-00712-9
64. Andre F, Bachelot T, Campone M, Dalenc F, Perez-Garcia JM, Hurvitz SA, Turner N, Rugo H, Smith JW, Deudon S, et al. Targeting FGFR with dovitinib (TKI258): preclinical and clinical data in breast cancer. Clin Cancer Res (2013) 19:3693–3702. doi: 10.1158/1078-0432.CCR-13-0190
65. Lv Y, Zhang H, Zhao Y, Zhang H, Wang T. A case report of low-dose apatinib in the treatment of advanced triple-negative breast cancer. Transl Breast Cancer Res (2024) 5:8. doi: 10.21037/tbcr-23-24
66. Zhuang C, Liu X, Song Y, He P, Zheng D, Yan X, Zhong X, Tian T, Wei B, Xie YX, et al. Camrelizumab plus apatinib and chemotherapy as neoadjuvant therapy for triple negative breast cancer (TNBC): A single-arm, prospective phase II study (MA-BC-II-026). JCO (2025) 43: doi: 10.1200/JCO.2025.43.16_suppl.e12623
67. Hu X, Cao J, Hu W, Wu C, Pan Y, Cai L, Tong Z, Wang S, Li J, Wang Z, et al. Multicenter phase II study of Apatinib in non-triple-negative metastatic breast cancer. BMC Cancer (2014) 14:820. doi: 10.1186/1471-2407-14-820
68. Kumari M, Krishnamurthy PT, Pinduprolu S kiran SS, Sola P. DR-5 and DLL-4 mAb Functionalized SLNs of Gamma-Secretase Inhibitors- An Approach for TNBC Treatment. Adv Pharm Bull (2021) 11:618–623. doi: 10.34172/apb.2021.070
69. Means-Powell JA, Mayer IA, Ismail-Khan R, Del Valle L, Tonetti D, Abramson VG, Sanders MS, Lush RM, Sorrentino C, Majumder S, et al. A Phase Ib Dose Escalation Trial of RO4929097 (a γ-secretase inhibitor) in Combination with Exemestane in Patients with ER + Metastatic Breast Cancer (MBC). Clin Breast Cancer (2022) 22:103–114. doi: 10.1016/j.clbc.2021.10.013
70. Wang Z-J, Zhan X-Y, Ma L-Y, Yao K, Dai H-Y, Kumar Santhanam R, Zhou M-S, Jia H. Activation of the γ-secretase/NICD-PXR/Notch pathway induces Taxol resistance in triple-negative breast cancer. Biochem Pharmacol (2024) 230:116577. doi: 10.1016/j.bcp.2024.116577.
71. Sardesai S, Badawi M, Mrozek E, Morgan E, Phelps M, Stephens J, Wei L, Kassem M, Ling Y, Lustberg M, et al. A phase I study of an oral selective gamma secretase (GS) inhibitor RO4929097 in combination with neoadjuvant paclitaxel and carboplatin in triple negative breast cancer. Invest New Drugs (2020) 38:1400–1410. doi: 10.1007/s10637-020-00895-5
72. Vigolo M, Urech C, Lamy S, Monticone G, Zabaleta J, Hossain F, Wyczechowska D, Del Valle L, O’Regan RM, Miele L, et al. The Efficacy of CB-103, a First-in-Class Transcriptional Notch Inhibitor, in Preclinical Models of Breast Cancer. Cancers (Basel) (2023) 15:3957. doi: 10.3390/cancers15153957
73. Yang R, Li Y, Wang H, Qin T, Yin X, Ma X. Therapeutic progress and challenges for triple negative breast cancer: targeted therapy and immunotherapy. Mol Biomed (2022) 3:8. doi: 10.1186/s43556-022-00071-6
74. Shastry M, Jacob S, Rugo HS, Hamilton E. Antibody-drug conjugates targeting TROP-2: Clinical development in metastatic breast cancer. Breast (2022) 66:169–177. doi: 10.1016/j.breast.2022.10.007
75. Jeon Y, Jo U, Hong J, Gong G, Lee HJ. Trophoblast cell-surface antigen 2 (TROP2) expression in triple-negative breast cancer. BMC Cancer (2022) 22:1014. doi: 10.1186/s12885-022-10076-7
76. Bardia A, Hurvitz SA, Tolaney SM, Loirat D, Punie K, Oliveira M, Brufsky A, Sardesai SD, Kalinsky K, Zelnak AB, et al. Sacituzumab Govitecan in Metastatic Triple-Negative Breast Cancer. N Engl J Med (2021) 384:1529–1541. doi: 10.1056/NEJMoa2028485
77. Traina TA, Miller K, Yardley DA, Eakle J, Schwartzberg LS, O’Shaughnessy J, Gradishar W, Schmid P, Winer E, Kelly C, et al. Enzalutamide for the Treatment of Androgen Receptor–Expressing Triple-Negative Breast Cancer. J Clin Oncol (2018) 36:884–890. doi: 10.1200/JCO.2016.71.3495
78. Walsh EM, Gucalp A, Patil S, Edelweiss M, Ross DS, Razavi P, Modi S, Iyengar NM, Sanford R, Troso-Sandoval T, et al. Adjuvant enzalutamide for the treatment of early-stage androgen-receptor positive, triple-negative breast cancer: a feasibility study. Breast Cancer Res Treat (2022) 195:341–351. doi: 10.1007/s10549-022-06669-2
79. Gucalp A, Tolaney S, Isakoff SJ, Ingle JN, Liu MC, Carey LA, Blackwell K, Rugo H, Nabell L, Forero A, et al. Phase II Trial of Bicalutamide in Patients with Androgen Receptor–Positive, Estrogen Receptor–Negative Metastatic Breast Cancer. Clin Cancer Res (2013) 19:5505–5512. doi: 10.1158/1078-0432.CCR-12-3327
80. Kong Y, Qu F, Yuan X, Yan X, Yu W. Effect of Bicalutamide on the proliferation and invasion of human triple negative breast cancer MDA-MB-231 cells. Medicine (Baltimore) (2020) 99:e19822. doi: 10.1097/MD.0000000000019822
